# Host RAB11FIP5 protein inhibits the release of Kaposi’s sarcoma-associated herpesvirus particles by promoting lysosomal degradation of ORF45

**DOI:** 10.1371/journal.ppat.1009099

**Published:** 2020-12-14

**Authors:** Xiaoqin Wei, Jiazhen Dong, Chin-Chen Cheng, Mingjun Ji, Lei Yu, Shengqiu Luo, Shuwen Wu, Lei Bai, Ke Lan

**Affiliations:** State Key Laboratory of Virology, College of Life Sciences, Wuhan University, Wuhan, China; University of Southern California, UNITED STATES

## Abstract

Open reading frame (ORF) 45 is an outer tegument protein of Kaposi’s sarcoma-associated herpesvirus (KSHV). Genetic analysis of an ORF45-null mutant revealed that ORF45 plays a key role in the events leading to the release of KSHV particles. ORF45 associates with lipid rafts (LRs), which is responsible for the colocalization of viral particles with the trans-Golgi network and facilitates their release. In this study, we identified a host protein, RAB11 family interacting protein 5 (RAB11FIP5), that interacts with ORF45 *in vitro* and *in vivo*. RAB11FIP5 encodes a RAB11 effector protein that regulates endosomal trafficking. Overexpression of RAB11FIP5 in KSHV-infected cells decreased the expression level of ORF45 and inhibited the release of KSHV particles, as reflected by the significant reduction in the number of extracellular virions. In contrast, silencing endogenous RAB11FIP5 increased ORF45 expression and promoted the release of KSHV particles. We further showed that RAB11FIP5 mediates lysosomal degradation of ORF45, which impairs its ability to target LRs in the Golgi apparatus and inhibits ORF45-mediated colocalization of viral particles with the trans-Golgi network. Collectively, our results suggest that RAB11FIP5 enhances lysosome-dependent degradation of ORF45, which inhibits the release of KSHV particles, and have potential implications for virology and antiviral design.

## Author summary

ORF45 plays a key role in events leading to viral particle release following KSHV reactivation; however, the regulation of this process is incompletely understood. We report the host RAB11FIP5 protein as a novel ORF45-binding protein. Direct interaction of RAB11FIP5 with ORF45 promotes the translocation of ORF45 to lysosomes for its degradation. Additionally, binding of RAB11FIP5 to ORF45 inhibits the colocalization of ORF45 with LRs in the Golgi, hence decreasing the colocalization of viral particles with the Golgi and inhibiting viral particle release. Our study revealed that RAB11FIP5 inhibits ORF45-mediated KSHV release by promoting ORF45 degradation and provides new insights for antiviral design.

## Introduction

Kaposi’s sarcoma-associated herpesvirus (KSHV) is etiologically associated with Kaposi’s sarcoma (KS), primary effusion lymphoma (PEL) and multicentric Castleman disease (MCD) [1–3]. KSHV virions exhibit an icosahedral nucleocapsid surrounded by a lipid bilayer envelope, and a tegument layer separates the capsid and envelope [4–6]. Like that of other herpesviruses, the life cycle of KSHV has two phases: latent infection and lytic replication [7]. During the latent infection phase, few viral genes are expressed, and no virus particles are produced [8,9]. KSHV can be reactivated from latency by various stimuli [10–12]. The lytic replication of KSHV, release of virions, and continual infection of fresh cells are crucial for its viral tumorigenicity and pathogenesis [13–16]. Despite the importance of lytic production in viral pathogenicity, the process of KSHV release is incompletely elucidated. A more complete understanding of KSHV release would benefit the identification of novel antiviral targets for KSHV-related diseases.

Herpesvirus tegument proteins contribute to diverse functions in the viral life cycle. These proteins play critical roles in modulating the host cellular environment, transporting capsids to the nucleus along microtubules following viral entry, participating in the complex chain of events involving viral assembly and egress, and other events [17–22]. Open reading frame (ORF) 45, an outer tegument protein of KSHV particles, has been demonstrated to be a multifunctional protein [23–27]. ORF45-null KSHV produces a much lower yield of progeny virions than wild-type KSHV [24]. ORF45 also regulates the process of KSHV particle egress by interacting with KIF3A [28]. ORF45 associates with lipid rafts (LRs) in the Golgi apparatus, mediating the colocalization of viral particles with the cytoplasmic trans-Golgi network and facilitating the release of viral particles into the supernatant [29].

Endocytosed proteins are either recycled back to the plasma membrane or transported to lysosomes for degradation [30–32]. The RAB11 GTPase has emerged as an important regulator of endocytic transport [33]. RAB11-mediated recycling endosomes can transport their cargo back to the plasma membrane or promote its degradation [34–38]. RAB11 family interacting proteins (RAB11FIPs) regulate RAB11-dependent endosomal recycling. In mammals, the RAB11FIP family comprises five proteins, all of which interact with RAB11 and homodimerize via a conserved C-terminal coiled-coil structure termed the Rab11 binding domain (RBD). These five RAB11FIP molecules are grouped into two classes: class I (RAB11FIP1/2/5), with N-terminal C2 domains; and class II (RAB11FIP3/4), with EF-hand domains [34]. A plethora of evidence suggests that RAB11-mediated endosomal recycling plays a critical role in the regulation of viral infection [39–43]. Previous study reported that vRNP of influenza virus interacts with RAB11, which is essential for the trafficking of vRNPs and subsequent efficient production of infectious virus [44–47]. RAB11FIP3 is essential for filamentous but not spherical virion formation [48]. The release of respiratory syncytial virus (RSV) has been reported to be independent of the ESCRT machinery but controlled by RAB11FIP1 and RAB11FIP2 [49,50]. RAB11FIP4 is important for the trafficking of human cytomegalovirus (HCMV) components [51]. However, RAB11FIP5 has not previously been linked to viral infection.

A global mapping study indicated that ORF45 may associate with RAB11FIP5 [52]. RAB11FIP5 encodes a RAB11 effector protein that regulates endosomal trafficking [34], and ORF45 has been shown to play a key role in KSHV release [24,28,29]. Therefore, we hypothesized that RAB11FIP5 may be involved in ORF45-mediated release of KSHV particles. To test this hypothesis, we first confirmed the interaction between ORF45 and RAB11FIP5. We then found that overexpression of RAB11FIP5 reduces the release of KSHV virions from the cytoplasm but does not affect viral replication and viral gene transcription. However, knockdown of RAB11FIP5 increased the release of KSHV virions. We further showed that RAB11FIP5 promotes the translocation of ORF45 to the lysosome and then accelerates its degradation. Moreover, RAB11FIP5 impaired the colocalization of ORF45 with LRs in the Golgi apparatus and prevented the translocation of KSHV particles to the Golgi. Taken together, the results of our study reveal that RAB11FIP5 serves as a negative regulator of ORF45 that can inhibit the release of KSHV particles.

## Results

### RAB11FIP5 interacts with KSHV ORF45

**Fig 1:**
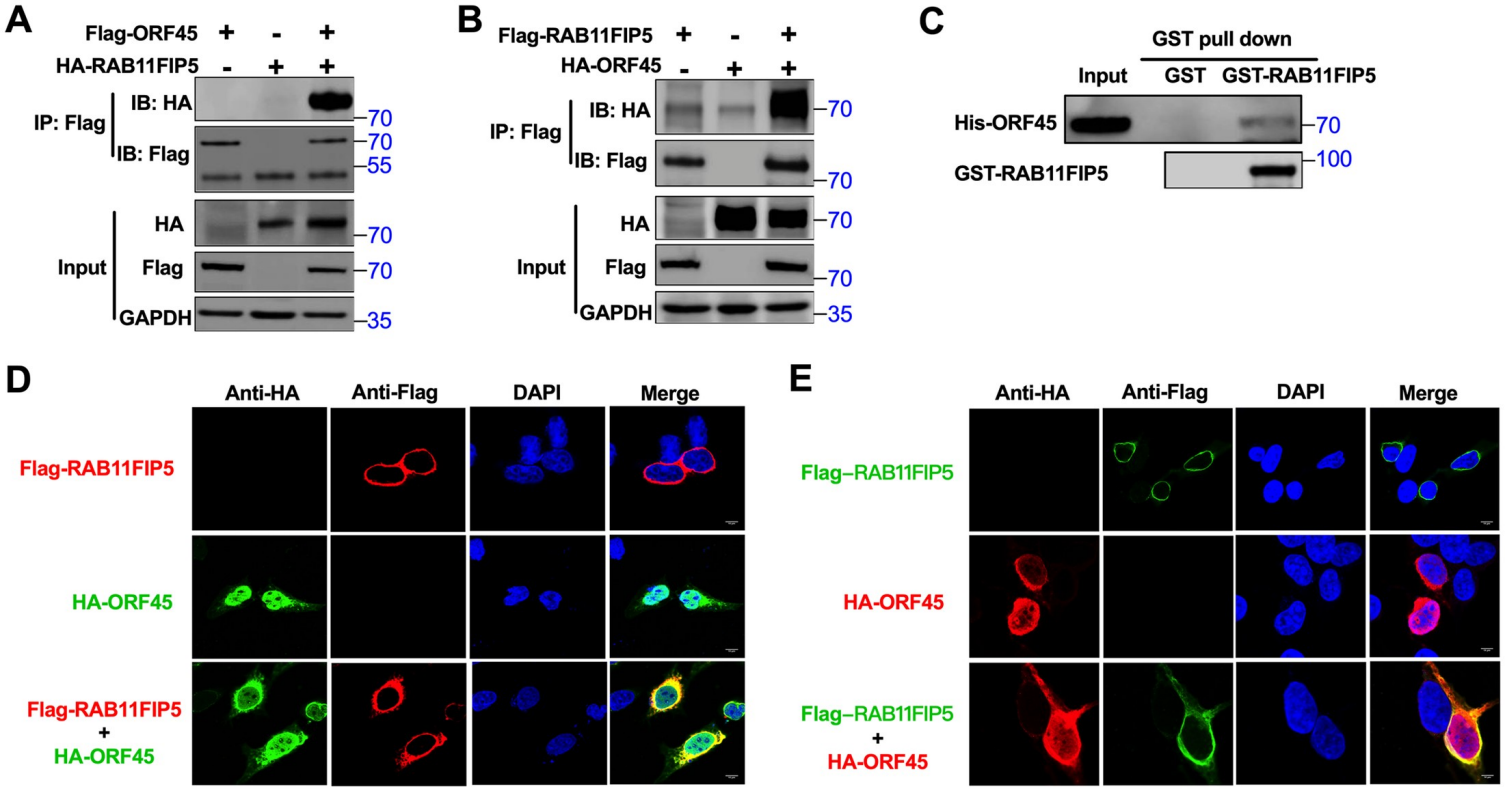
RAB11FIP5 interacts with KSHV ORF45. (A) HEK293T cells were transfected with Flag-ORF45 alone, with HA-RAB11FIP5 alone or with both Flag-ORF45 and HA-RAB11FIP5. Cell lysates were immunoprecipitated with an anti-Flag antibody and were then analyzed by western blotting with the indicated antibodies. (B) HEK293T cells were transfected with Flag-RAB11FIP5 alone, with HA-ORF45 alone or with both Flag-RAB11FIP5 and HA-ORF45. Cell lysates were immunoprecipitated with an anti-Flag antibody and were then analyzed by western blotting with the indicated antibodies. (C) *In vitro* GST affinity binding assay. Bacterially expressed GST and GST-RAB11FIP5 bound to GST-Sepharose beads were incubated with purified His-tagged ORF45, and the pulled down lysates were immunoblotted with anti-His or anti-GST antibodies. Colocalization of RAB11FIP5 and ORF45 in HeLa cells (D) and HEK293T cells (E). After transfection with Flag-RAB11FIP5 and HA-ORF45, HeLa cells and HEK293T cells were fixed with 4% paraformaldehyde and were then labeled with anti-HA and anti-Flag antibodies. FITC- and Cy3-conjugated secondary antibodies were used to visualize the labeled RAB11FIP5 and ORF45 proteins, respectively. DAPI was used to label cell nuclei.

A global mapping study that identified interacting partners of 89 KSHV proteins was previously reported. In this study, RAB11FIP5 was shown to be a potential interacting partner of KSHV ORF45 [52]. To confirm the interaction between ORF45 and RAB11FIP5, HEK293T cells were transfected with Flag-tagged ORF45 and HA-tagged RAB11FIP5 individually or together. RAB11FIP5 was coimmunoprecipitated with ORF45 (Fig 1A). The reverse coimmunoprecipitation (Co-IP) experiment also showed that the ORF45 protein was specifically coimmunoprecipitated with RAB11FIP5 (Fig 1B). To determine whether this interaction is direct, we performed an *in vitro* binding assay. *In vitro*-purified His-tagged ORF45 protein was incubated with purified GST or GST-fused RAB11FIP5 beads, and ORF45 was found to interact directly with RAB11FIP5 (Fig 1C).

To verify the above results of the immunoprecipitation and *in vitro* binding assays, we performed immunofluorescence analysis (IFA) to determine whether RAB11FIP5 and ORF45 can be colocalized to the same cellular compartment. HeLa cells and HEK293T cells were transiently cotransfected with Flag-tagged RAB11FIP5 and HA-tagged ORF45. RAB11FIP5 and ORF45 were colocalized in the same cytoplasmic compartment in both HeLa and HEK293T cells (Fig 1D and 1E). These results suggest that exogenously transfected RAB11FIP5 and ORF45 proteins are colocalized in the cytoplasm.

**Fig 2:**
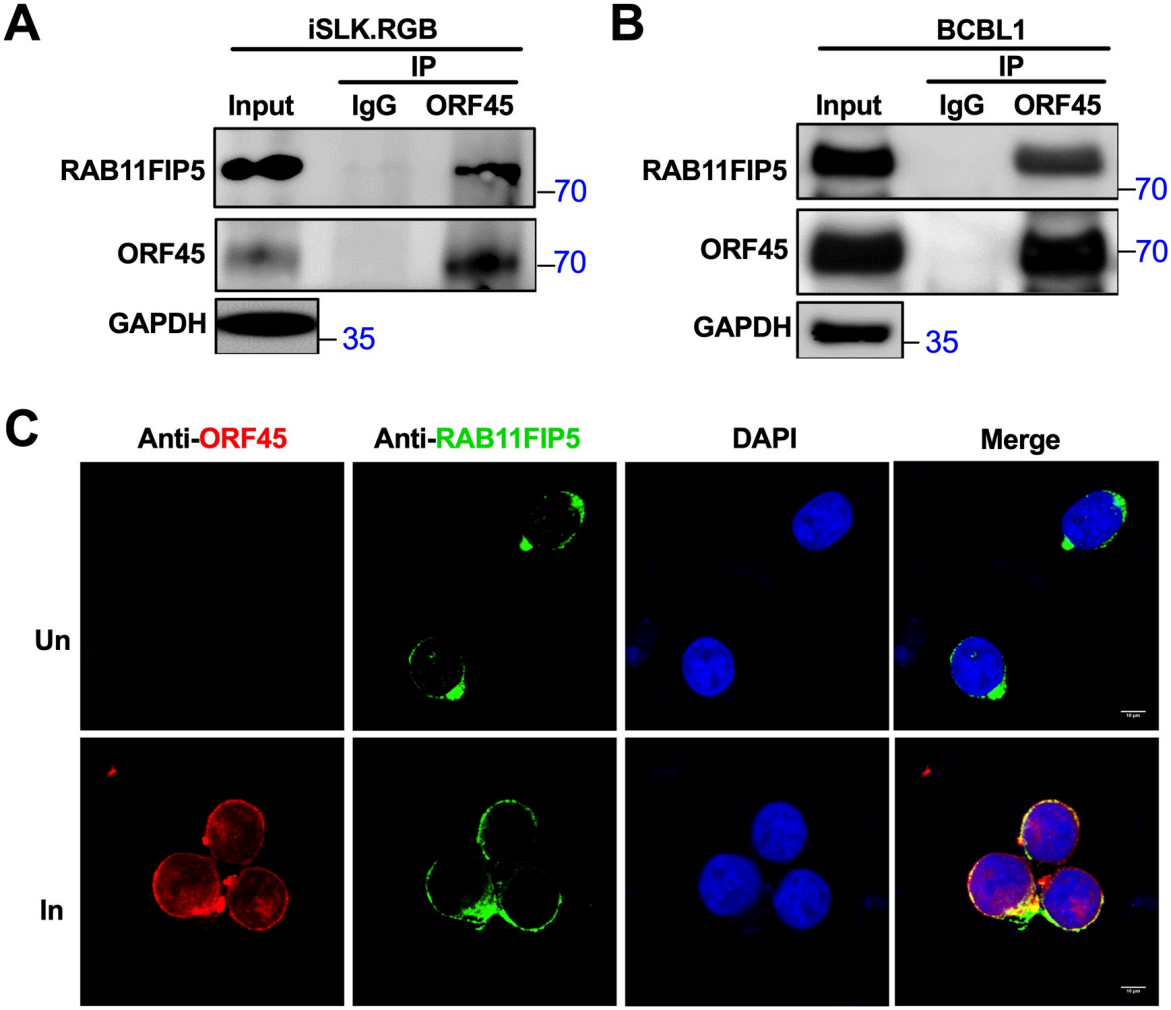
The interaction between endogenous RAB11FIP5 and ORF45. (A) Co-IP of endogenous ORF45 and RAB11FIP5 in KSHV-positive iSLK.RGB cells. Lytic replication of KSHV in the cells was induced by dox, and cell lysates were subjected to immunoprecipitation with the anti-ORF45 antibody or mouse IgG control antibody. Purified proteins, along with input samples, were subjected to western blotting with the indicated antibodies. (B) Co-IP of endogenous ORF45 and RAB11FIP5 in KSHV-positive BCBL1 cells. Lytic replication of KSHV in the cells was induced by VPA, and the cells were treated as described in (A). (C) Endogenous RAB11FIP5 colocalized with endogenous ORF45 in the cytoplasm. BCBL1 cells uninduced (Un) or induced with VPA (In) were fixed and labeled with anti-RAB11FIP5 and anti-ORF45 antibodies and were then incubated with FITC- or Cy3-conjugated secondary antibodies.

To verify the interaction between endogenous RAB11FIP5 and ORF45, we carried out Co-IP with KSHV-infected iSLK.RGB and BCBL1 cell lines that harbored latent KSHV episomes. After the cells were induced by doxycycline (dox) (iSLK.RGB) or treated with valproic acid (VPA) (BCBL1) for 24 h to activate the expression of endogenous ORF45, cell lysates were immunoprecipitated with anti-ORF45 or IgG control antibodies. As expected, endogenous RAB11FIP5 was associated with the ORF45 protein in KSHV-infected cells (Fig 2A and 2B). We also performed IFA to explore whether endogenous ORF45 and RAB11FIP5 can be colocalized in similar cytoplasmic compartments in BCBL1 cells naturally infected with KSHV. Twenty-four hours after induction by VPA, cells were fixed for IFA, probed with anti-ORF45 as well as anti-RAB11FIP5 antibodies, and finally incubated with appropriate secondary antibodies. Endogenous RAB11FIP5 and ORF45 were colocalized in the same cytoplasmic compartments in BCBL1 cells (Fig 2C). We also observed the colocalization of RAB11FIP5 and ORF45 in the cytoplasmic compartment of iSLK-BAC16 cells (S1 Fig).

Taken together, these results confirm that the host RAB11FIP5 protein is a novel ORF45-interacting protein.

### Mapping the interaction domains in RAB11FIP5 and ORF45

**Fig 3:**
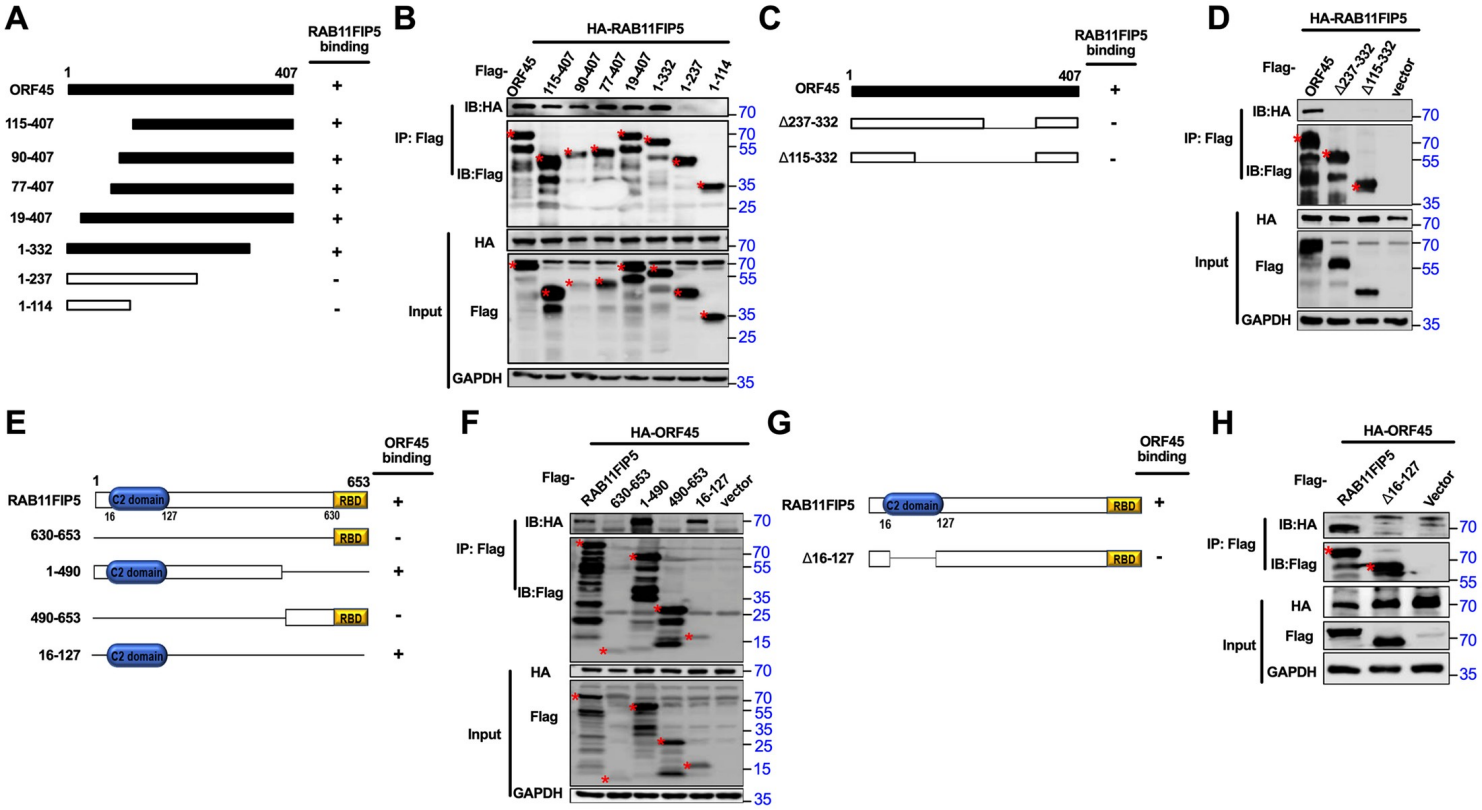
Mapping the interaction domains in ORF45 and RAB11FIP5. (A) Schematics of the ORF45 truncation mutants, including 115–407 (aa 115-aa 407), 90–407 (aa 90-aa 407), 77–407 (aa 77-aa 407), 19–407 (aa 19- aa 407), 1–332 (aa 1- aa 332), 1–237 (aa 1- aa 237), and 1–114 (aa 1- aa 114), are shown. (B) Defining the RAB11FIP5-interacting domain in ORF45. Co-IP and western blotting of HEK293T cells cotransfected with HA-tagged RAB11FIP5 and a vector expressing the indicated Flag-tagged ORF45 truncations or full-length ORF45. (C) Schematic diagram of ORF45 deletion mutants (Δ237–332 and Δ115–332). (D) Co-IP and western blotting of HEK293T cells cotransfected with HA-RAB11FIP5 and Flag-tagged ORF45 deletion mutants. (E) Schematics of RAB11FIP5 truncation mutants, including 630–653 (aa 630- aa 653), 1–490 (aa 1- aa 490), 490–653 (aa 490- aa 653), and 16–127 (aa 16- aa 127), are shown. (F) Defining the ORF45-interacting domain in RAB11FIP5. Co-IP and western blotting of HEK293T cells cotransfected with HA-tagged ORF45 and the indicated Flag-tagged RAB11FIP5 truncations or full-length RAB11FIP5. An empty vector was used as the negative control. (G) Schematic diagram of a C2 region deletion mutant of RAB11FIP5 (Δ16–127). (H) Co-IP and western blotting of HEK293T cells cotransfected with HA-ORF45 and the Flag-tagged C2 region deletion mutant of RAB11FIP5.

To map the individual ORF45 regions responsible for the interaction with RAB11FIP5, we generated a series of Flag-tagged ORF45 deletion mutants [28] (Fig 3A). HEK293T cells were cotransfected with RAB11FIP5 and wild-type or mutant ORF45. The region of ORF45 comprising amino acid (aa) residues 237–332 was found to be required for its interaction with RAB11FIP5 (Fig 3B). In addition, deletion of aa residues 237 to 332 abolished the interaction of ORF45 with RAB11FIP5 (Fig 3C and 3D).

A similar approach was employed to determine the minimum region in RAB11FIP5 required for its interaction with ORF45. A series of Flag-tagged RAB11FIP5 truncation mutants were generated, and the ability of these mutants to interact with ORF45 was assessed (Fig 3E). As shown in Fig 3F, ORF45 coimmunoprecipitated with the RAB11FIP5 truncation mutants containing the C2 domain (comprising aa residues 16–127). However, deletion of the C2 domain abolished the interaction of RAB11FIP5 with ORF45 (Fig 3G and 3H).

### RAB11FIP5 inhibits the release of KSHV particles

**Fig 4:**
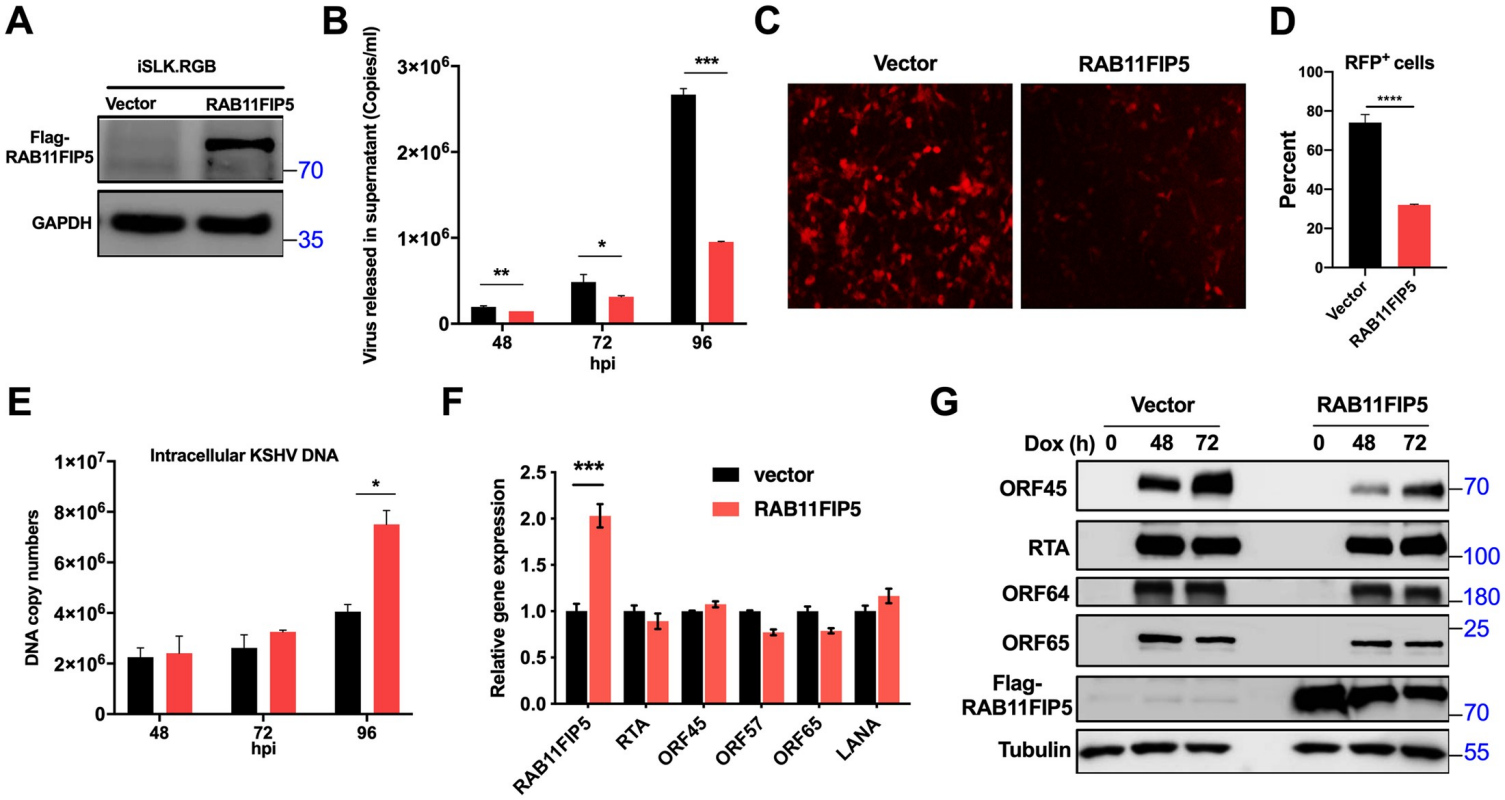
Overexpression of RAB11FIP5 inhibits the release of progeny virus. (A) iSLK.RGB cells were stably transduced with lentiviruses containing a Flag-tagged RAB11FIP5 expression plasmid or an empty vector plasmid and were named iSLK.RGB-RAB11FIP5 or iSLK.RGB-Vector cells, respectively. Overexpression of RAB11FIP5 was detected by western blotting. (B) iSLK.RGB-Vector and iSLK.RGB-RAB11FIP5 cells were treated with dox for different time points as indicated. Extracellular virions were collected from the culture medium and treated with DNase I. Viral DNA was extracted, and KSHV genomic DNA copy numbers were estimated by qPCR by comparison with external standards containing known concentrations of the viral K9 plasmid. (C) Supernatants (500 μl) collected from dox-induced iSLK.RGB-Vector and iSLK.RGB-RAB11FIP5 cells at 72 hpi were incubated with HEK293T cells. The infection rate of HEK293T cells was assessed by fluorescence microscopy. (D) Flow cytometry analysis and quantitation of the percentage of RFP^+^ cells from (C). (E) Intracellular KSHV genomic DNA was extracted from harvested cells and quantified by qPCR with normalization to GAPDH. (F) RNA was extracted from dox-induced iSLK.RGB-Vector and iSLK.RGB-RAB11FIP5 cells at 72 hpi to measure the transcription level of several KSHV genes: RTA, ORF45, ORF57, ORF65 and LANA. (G) Lysates from dox-induced iSLK.RGB-Vector and iSLK.RGB-RAB11FIP5 cells were analyzed by western blotting at the indicated time points. The expression levels of several KSHV proteins, including ORF45, RTA, ORF64 and ORF65, were determined by immunoblotting with the indicated antibodies.

ORF45 was found to be expressed as an immediate-early protein during lytic replication [53]. We thus sought to determine whether RAB11FIP5 plays a role in the regulation of the KSHV lytic replication cycle by interacting with the ORF45 protein. To this end, we constructed two stable cell lines by transducing the KSHV-infected iSLK.RGB cell line with either a lentiviral vector expressing Flag-tagged RAB11FIP5 or an empty vector as the control, yielding the iSLK.RGB-RAB11FIP5 and iSLK.RGB-Vector cell lines, respectively (Fig 4A). Lytic replication of KSHV in these two cell lines was activated by dox-induced RTA expression [54–56]. Overexpression of RAB11FIP5 resulted in a significant reduction in the release of KSHV virions compared to that in the control group (Fig 4B). In addition, progeny viruses prepared from cultured iSLK.RGB-RAB11FIP5 and iSLK.RGB-Vector cells in the same volume of cell culture supernatant were used to infect HEK293T cells, and the intensity of red fluorescence indicating KSHV-infected cells was reduced in the RAB11FIP5-overexpressing group (Fig 4C). To further confirmed this result, the infection rate of HEK293T cells was analyzed by flow cytometry, and we found that the percentage of RFP^+^ cells in the RAB11FIP5-overexpressing group was reduced (Fig 4D). However, RAB11FIP5 overexpression did not affect KSHV DNA replication (Fig 4E) or the transcription levels of viral genes (Fig 4F). Interestingly, at 96 h after dox treatment, the abundance of intracellular KSHV genomic DNA in RAB11FIP5-overexpressing cells appeared to be higher than that in control cells, probably resulting from failure to release progeny virus particles into the supernatant, thus allowing intracellular accumulation of viral genomic DNA (Fig 4E). Furthermore, RAB11FIP5 overexpression did not affect the protein expression levels of RTA, ORF64 and ORF65 but significantly decreased that of ORF45 compared with the corresponding expression levels in control cells (Fig 4G). However, the RAB11FIP5 C2 domain deletion mutant (Δ16–127) that cannot bind to ORF45 had no significant effect on the release of KSHV virions (S2 Fig).

**Fig 5:**
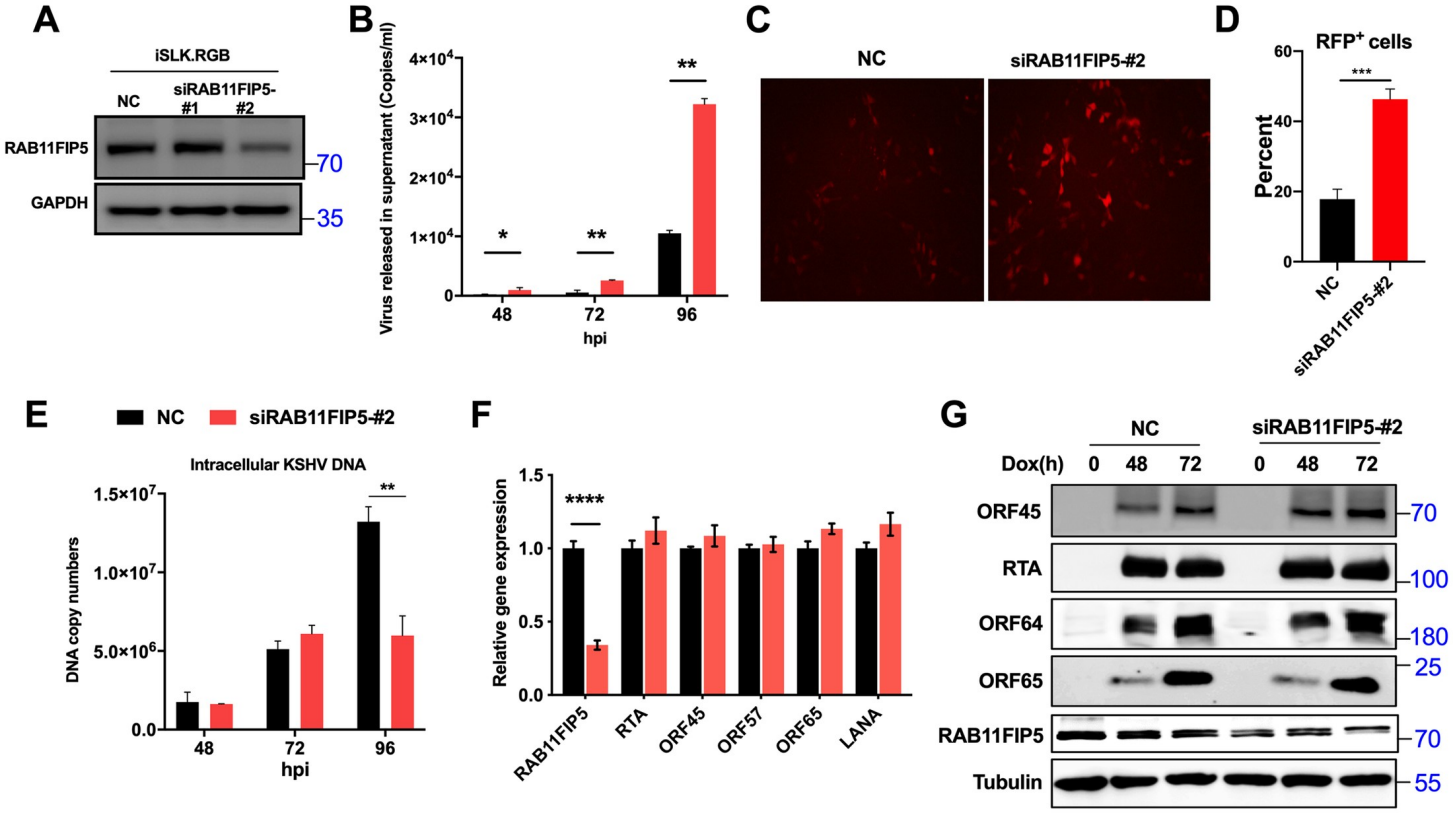
Knockdown of endogenous RAB11FIP5 promotes the release of progeny virus. (A) iSLK.RGB cells were transfected with control siRNA and two RAB11FIP5-specific siRNAs (#1 and #2). The knockdown efficiency was determined by western blotting. (B) iSLK.RGB cells were transfected with control siRNA and siRAB11FIP5-#2. Twenty-four hours after transfection, cells were induced with dox for different time points as indicated. Extracellular virions were collected from the culture medium and treated with DNase I. Viral DNA was extracted, and KSHV genomic DNA copy numbers were estimated by qPCR by comparison with external standards containing known concentrations of the viral K9 plasmid. (C) Supernatants (500 μl) collected from dox-induced cells at 72 hpi were incubated with HEK293T cells. The infection rate of HEK293T cells was examined by fluorescence microscopy. (D) Flow cytometry analysis and quantitation of the percentage of RFP^+^ cells from (C). (E) Intracellular KSHV genomic DNA was extracted from harvested cells and quantified by qPCR with normalization to GAPDH. (F) The transcription levels of KSHV genes were measured at 72 hpi. (G) The expression levels of several KSHV proteins were determined by immunoblotting at the indicated time post induction.

To further confirm the effect of RAB11FIP5 on the release of KSHV particles, iSLK.RGB cells were transfected separately with two RAB11FIP5-specific siRNAs (siRAB11FIP5-#1 and siRAB11FIP5-#2) or an equal amount of control siRNA for 24 h before induction with dox. siRAB11FIP5-#2 efficiently knocked down the expression of RAB11FIP5, and this siRNA was selected for subsequent experiments (Fig 5A). Indeed, knockdown of RAB11FIP5 significantly increased the release of KSHV virions compared to that in the control group (Fig 5B). In addition, HEK293T cells were infected with progeny virus particles prepared from cultured cells in the same volume, and the intensity of red fluorescence indicating successful infection was increased in the RAB11FIP5 knockdown group compared with the control group (Fig 5C). We also observed the infection rate of HEK293T cells was analyzed by flow cytometry and found that the RFP^+^ cells were increased in the RAB11FIP5 knockdown group (Fig 5D). Moreover, knockdown of RAB11FIP5 did not affect KSHV DNA replication (Fig 5E) or the transcription levels of viral genes (Fig 5F). At 96 h after dox treatment, the abundance of intracellular KSHV genomic DNA in RAB11FIP knockdown cells appeared to be lower than that in control cells, probably due to the release of an increased number of progeny virus particles into the supernatant (Fig 5E). Interestingly, we also found that knockdown of RAB11FIP increased the expression level of ORF45 but did not affect those of other viral genes (Fig 5G).

To strengthen these results, we used another KSHV-positive cell line BCBL1 to establish the BCBL1-RAB11FIP5 cell line and the BCBL1-Vector cell line (S3A Fig), and then treated these cells with VPA. Similar results were obtained from BCBL1 cells. Overexpression of RAB11FIP5 inhibited the release of KSHV virions and decreased the expression level of ORF45 (S3B and S3C Fig). Knockdown of RAB11FIP5 in BCBL1 by transfection with siRAB11FIP5-#2 (S3D Fig), which promoted the release of KSHV virions and increased the expression level of ORF45 (S3E and S3F Fig).

Collectively, these results demonstrate that RAB11FIP5 inhibits the process of KSHV particle release by targeting ORF45, possibly because of the reduced ORF45 protein expression level.

### RAB11FIP5 promotes lysosomal degradation of ORF45

**Fig 6:**
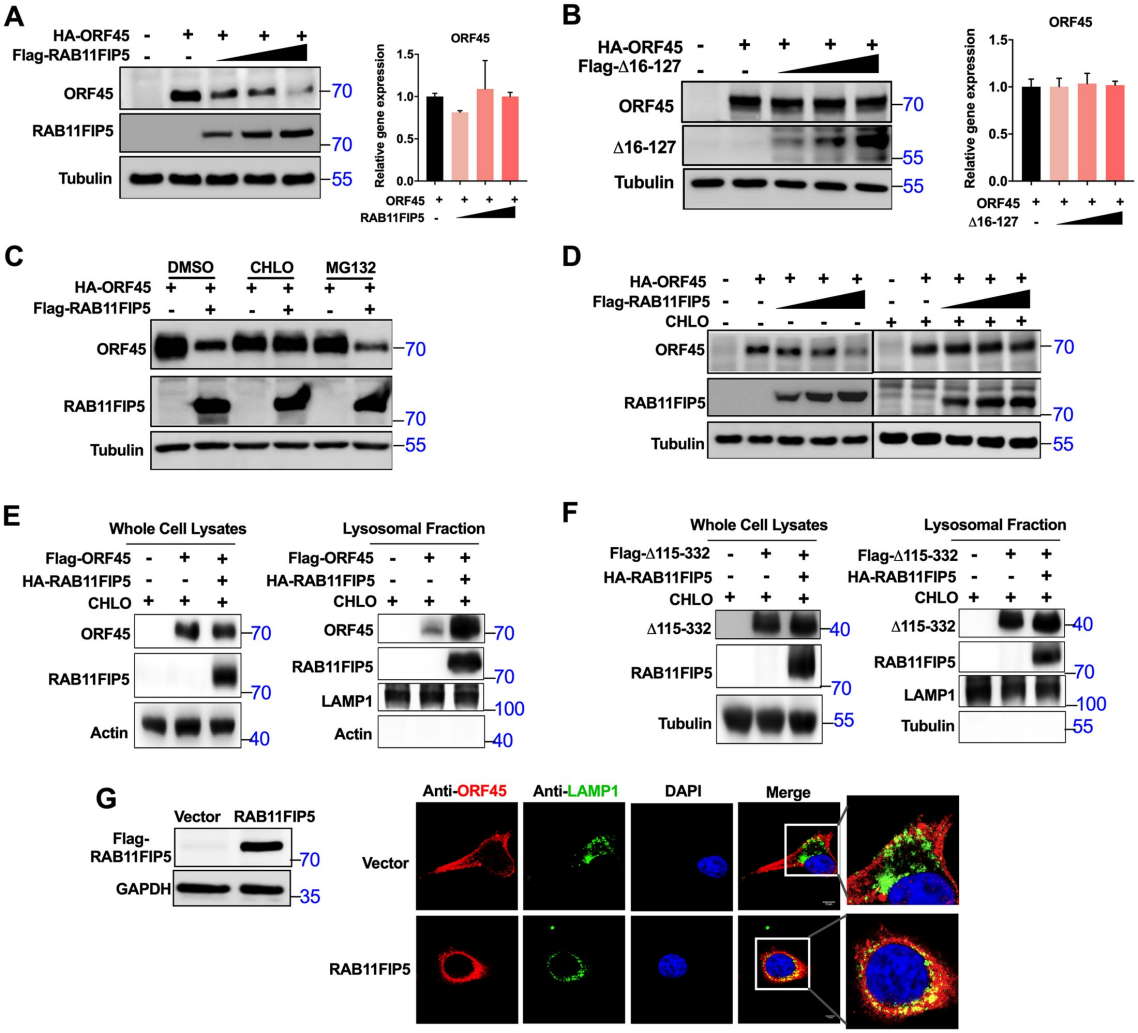
RAB11FIP5 promotes lysosomal degradation of ORF45. (A) Effect of RAB11FIP5 on ORF45 expression. HEK293T cells were cotransfected with 1 μg of ORF45 expression plasmid and increasing amounts of RAB11FIP5 expression vector (0, 0.5, 1, and 2 μg). ORF45 protein expression was assessed by immunoblotting with the indicated antibodies. ORF45 mRNA was detected using RT-qPCR with the indicated primers. (B) Effect of the RAB11FIP5 C2 domain deletion mutant (Δ16–127) on ORF45 expression. HEK293T cells were cotransfected with 1 μg of ORF45 expression plasmid and increasing amounts of the Δ16–127 expression vector (0, 0.5, 1, and 2 μg). ORF45 protein expression was assessed by immunoblotting. ORF45 mRNA was detected using RT-qPCR with the indicated primers. (C) Effects of inhibitors on RAB11FIP5-mediated destabilization of ORF45. HEK293T cells were transfected with the indicated plasmids. Fourteen hours after transfection, cells were treated with the indicated inhibitors for 6 h before immunoblot analysis was performed. (D) HEK293T cells were transiently cotransfected with HA-ORF45 and increasing amounts of Flag-RAB11FIP5 (0, 0.5, 1, and 2 μg). ORF45 protein expression was examined by western blotting in the presence and absence of CHLO. (E) HEK293T cells were transfected with Flag-ORF45 alone or cotransfected with Flag-ORF45 and HA-RAB11FIP5 for 36 h and were then treated with CHLO for another 6 h. A portion of cell samples for whole cell lysis was subjected to western blotting with the indicated antibodies (left panel). The lysosomal fraction was isolated from the remaining cell samples and subjected to western blotting with the indicated antibodies (right panel). (F) HEK293T cells were transfected with ORF45 deletion mutant Flag-Δ115–332 alone or cotransfected with Flag-Δ115–332 and HA-RAB11FIP5 for 36 h and were then treated with CHLO for another 6 h. A portion of cell samples for whole cell lysis was subjected to western blotting with the indicated antibodies (left panel). The lysosomal fraction was isolated from the remaining cell samples and subjected to western blotting with the indicated antibodies (right panel). (G) Stable clones of HeLa-RAB11FIP5 and HeLa-Vector cells were isolated and expanded from the monoclonal cell population by using the limiting dilution method. The expression of RAB11FIP5 was confirmed by western blotting. To assess the effect of RAB11FIP5 on the lysosomal localization of ORF45, stable clones of HeLa-Vector and HeLa-RAB11FIP5 cells were transfected with the ORF45 plasmid. Twenty-four hours after transfection, cells were treated with CHLO for 6 h and were then fixed with 4% paraformaldehyde. Double-label IFA was performed with mouse anti-ORF45 and rabbit anti-LAMP1 antibodies. FITC- and Cy3-conjugated secondary antibodies were used to visualize the labeled LAMP1 and ORF45 proteins, respectively. Images of the colocalization sites were enlarged as shown.

To evaluate whether RAB11FIP5 can regulate ORF45 protein expression, increasing amounts of RAB11FIP5 were coexpressed with a constant amount of ORF45 in HEK293T cells, and the effect of RAB11FIP5 on ORF45 expression was examined by western blot analysis. ORF45 expression decreased dose-dependently in the presence of RAB11FIP5 (Fig 6A). However, no significant changes in the ORF45 mRNA level were observed between cells with and without expression of RAB11FIP5 (Fig 6A). However, the RAB11FIP5 C2 domain deletion mutant (Δ16–127), which cannot bind to ORF45, completely lost the ability to downregulate ORF45 expression (Fig 6B). In addition, the lysosomal inhibitor chloroquine (CHLO) but not the proteasome inhibitor MG132 abolished RAB11FIP5-mediated downregulation of ORF45 expression (Fig 6C). CHLO treatment diminished the ability of RAB11FIP5 to inhibit the expression of ORF45 (Fig 6D). These results suggest that RAB11FIP5 may promote lysosome-dependent degradation of ORF45.

RAB11FIP5 plays an important role in endosomal recycling by recycling proteins back to the plasma membrane or transporting proteins to lysosomes for degradation [30–32,35]. To determine whether RAB11FIP5 induces ORF45 degradation by promoting the translocation of ORF45 to lysosomes, we isolated lysosomes from HEK293T cells transfected with ORF45 alone or cotransfected with ORF45 and RAB11FIP5; both groups of cells were treated with CHLO to inhibit ORF45 protein degradation. The result showed that RAB11FIP5 overexpression promoted the translocation of ORF45 to lysosomes (Fig 6E). However, overexpression of RAB11FIP5 did not affect the lysosomal translocation of the ORF45 deletion mutant Δ115–332 that cannot bind to RAB11FIP5 (Fig 6F). These results suggest that the interaction between RAB11FIP5 and ORF45 is necessary for RAB11FIP5-mediated degradation of ORF45.

To further confirm above result, stable clones of HeLa-RAB11FIP5 and HeLa-Vector cells were isolated and expanded from the monoclonal cell population by the limiting dilution method (Fig 6G). These two cell lines were transfected with an ORF45 expression plasmid. Confocal microcopy analysis indicated that a fraction of ORF45 localized in lysosomes in HeLa-Vector cells; however, an increased amount of ORF45 localized in lysosomes in HeLa-RAB11FIP5 cells (Fig 6G), indicating that RAB11FIP5 expression promotes the translocation of ORF45 to lysosomes.

### RAB11FIP5 impairs the colocalization of ORF45 with LRs

**Fig 7:**
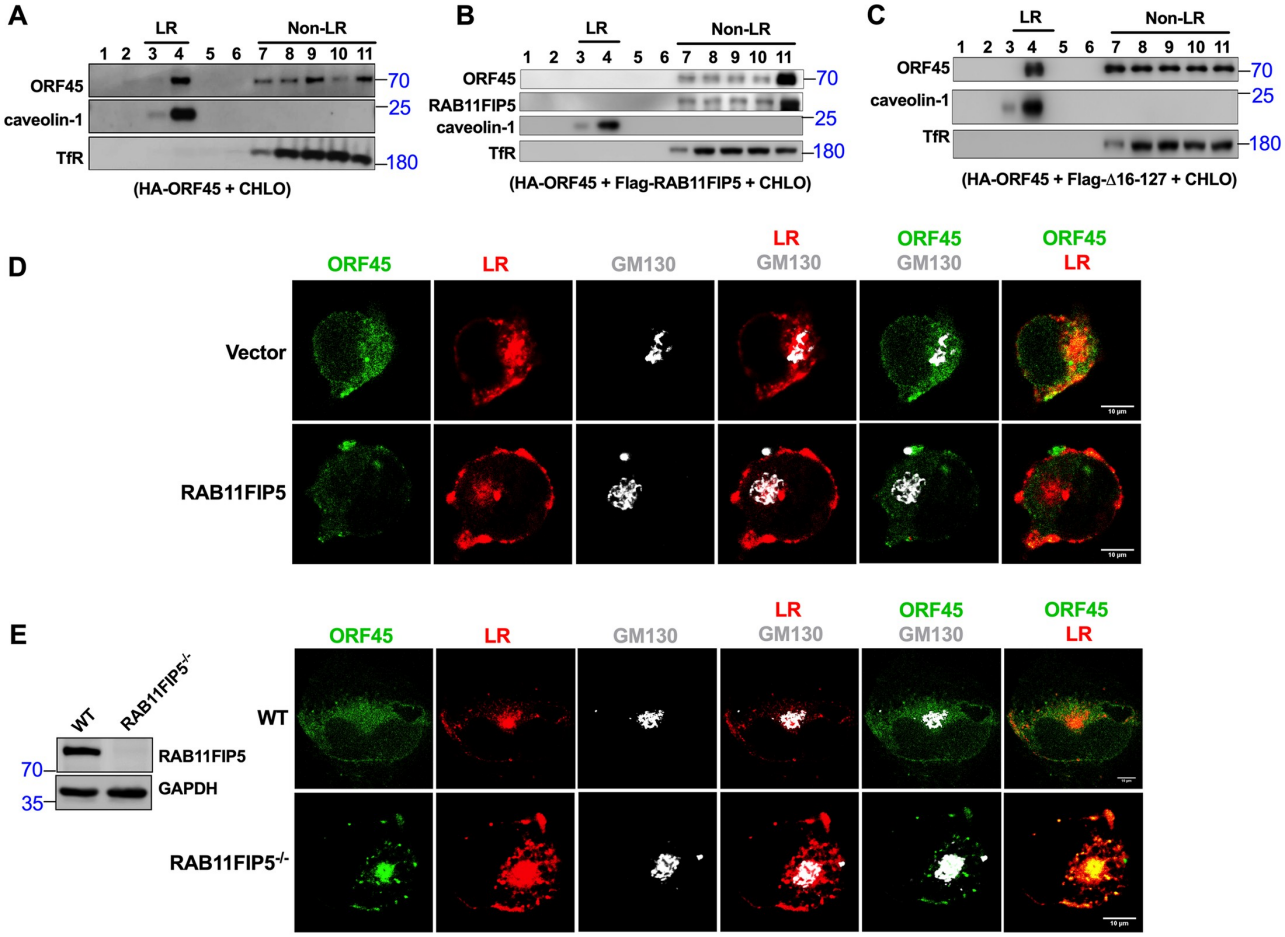
RAB11FIP5 inhibits ORF45 colocalization with LRs. (A) HEK293T cells were cotransfected with HA-ORF45 and Flag empty vector for 48 h and were then treated with CHLO for another 6 h. The cells were subjected to the membrane flotation assays as described in the Materials and Methods section. Eleven fractions (lanes 1–11) were collected (top to bottom) from samples subjected to sucrose gradient ultracentrifugation and analyzed by western blotting with specific antibodies as indicated. Caveolin-1 and TfR served as controls that defined the LR (lanes 3–4) and non-LR (lanes 7–11) fractions, respectively. (B) HEK293T cells were cotransfected with HA-ORF45 and Flag-RAB11FIP5 for 48 h and were then treated with CHLO for another 6 h. The cells were subjected to membrane flotation assays as described above. (C) HEK293T cells were cotransfected with HA-ORF45 and RAB11FIP5 C2 domain deletion mutant Flag-Δ16–127 for 48 h and were then treated with CHLO for another 6 h. The cells were subjected to the membrane flotation assays as described above. (D) Stable clones of HeLa-Vector and HeLa-RAB11FIP5 cells were transfected with the ORF45 plasmid. Twenty-four hours after transfection, the cells were treated with CHLO for 6 h and were then incubated with CTB-555 at 37°C for 30 min. Triple-label IFA was performed using CTB-555, rabbit polyclonal anti-GM130 and mouse monoclonal anti-ORF45 antibodies as described in the Materials and Methods section. Alexa Fluor 488-conjugated anti-mouse IgG (green) and Alexa Fluor 647-conjugated anti-rabbit IgG (white) were used as the corresponding secondary antibodies. (E) Stable clones of HeLa-WT and HeLa-RAB11FIP5^-/-^ cells were isolated and expanded from the monoclonal cell population by using the limiting dilution method. RAB11FIP5 knockout was confirmed by western blotting. Stable clones of HeLa-WT and HeLa-RAB11FIP5^-/-^ cells were transfected with the ORF45 plasmid. Twenty-four hours after transfection, the cells were treated with CHLO for 6 h and were then incubated with CTB-555 at 37°C for 30 min. Triple-label IFA was performed as described above.

Previously, ORF45 has been shown to be associated with LRs in the Golgi apparatus, promoting the colocalization of viral particles with trans-Golgi network and facilitating the release of viral particles. ORF45 mutation facilitates the colocalization of ORF45 with lysosomes and abolishes its association with LRs, which inhibits the release of viral particles [29]. As shown above, RAB11FIP5 expression promoted lysosome-dependent degradation of ORF45 and inhibited the release of viral particles. Thus, we reasoned that RAB11FIP5 expression affects the association between ORF45 and LRs in the Golgi apparatus. To test this hypothesis, we used a membrane flotation assay to examine the effect of RAB11FIP5 on the association between ORF45 and LRs [29]. This assay allows the isolation of detergent-resistant LRs and the detection of LR-associated proteins. HEK293T cells were transfected with HA-ORF45 alone or in combination with HA-ORF45 and Flag-RAB11FIP5, and both groups of cells were treated with CHLO to inhibit ORF45 protein degradation. Total cell lysates were fractionated by sucrose gradient ultracentrifugation, and eleven layers were obtained and analyzed by western blotting. Similar to the findings in a previous report [29], ORF45 was detected in the LR fractions containing the LR marker caveolin-1 (Fig 7A). Overexpression of RAB11FIP5 inhibited the ability of ORF45 to interact with LRs (Fig 7B). However, Overexpression of RAB11FIP5 mutant Δ16–127 had no significant effect on the colocalization of ORF45 with LRs (Fig 7C). To further confirm these results, we isolated and expanded stable clones of HeLa-WT and HeLa-RAB11FIP5^-/-^ cells (Fig 7E). HeLa-Vector, HeLa-RAB11FIP5, HeLa-WT and HeLa-RAB11FIP5^-/-^ cells were transfected with the ORF45 plasmid. The subcellular localization of ORF45 relative to LRs and the Golgi apparatus (GM130) was examined by triple-label IFA. As shown in Fig 7D, RAB11FIP5 overexpression decreased the colocalization of ORF45 with LRs in the Golgi apparatus. However, knockout of RAB11FIP5 significantly enhanced the colocalization of ORF45 with LRs in the Golgi apparatus (Fig 7E). Taken together, these data demonstrate that RAB11FIP5 prevents the localization of ORF45 to LRs.

### RAB11FIP5 impairs the transport of KSHV particles to the trans-Golgi network

**Fig 8:**
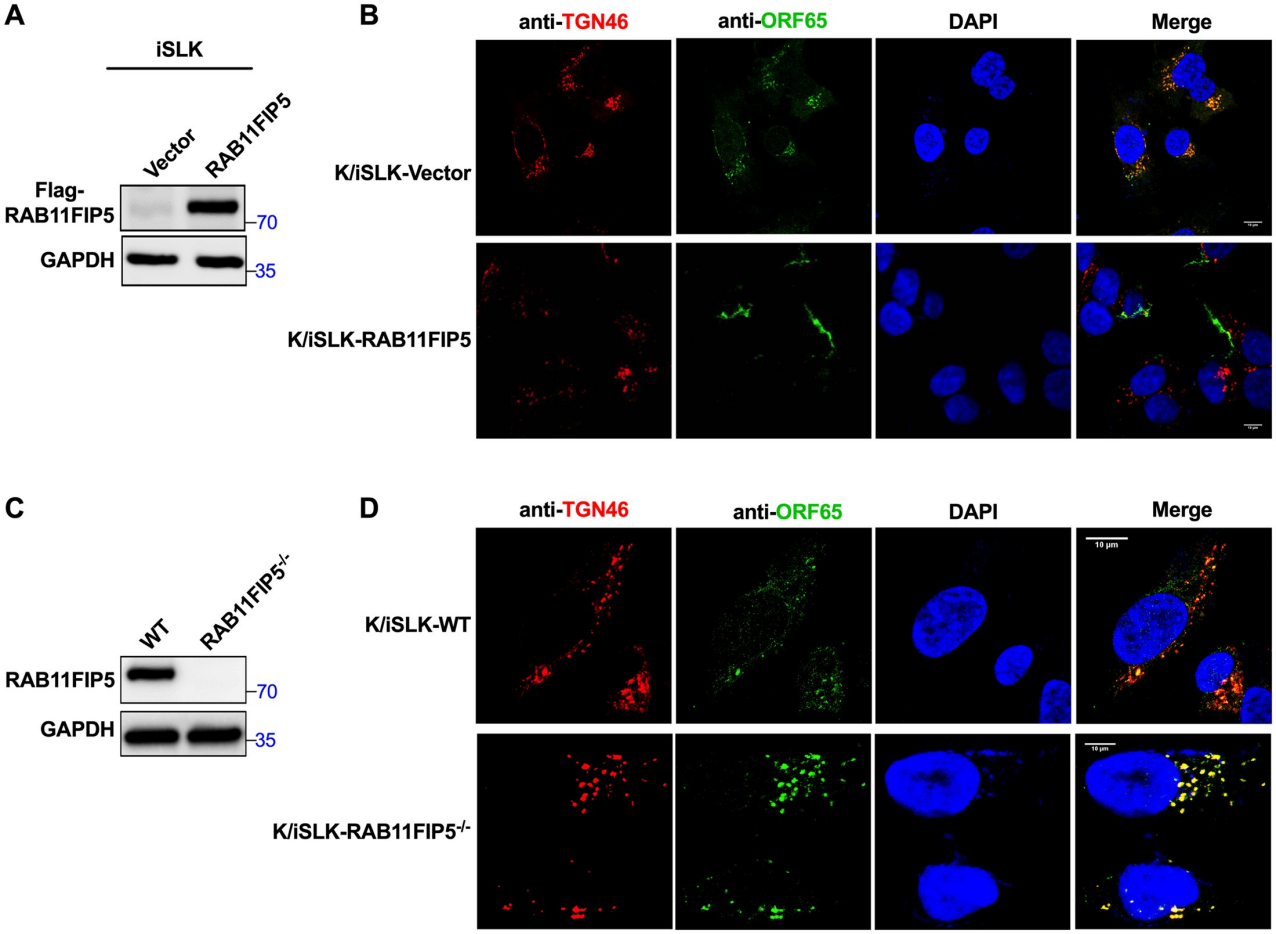
RAB11FIP5 impairs the translocation of KSHV particles to the trans-Golgi network. Stable clones of iSLK-Vector, iSLK-RAB11FIP5, iSLK-WT and iSLK-RAB11FIP5^-/-^ cells were isolated and expended from the monoclonal cell population by the limiting dilution method (A and C). These cells were infected with stock KSHV isolated from BCBL1 cells. Seventy-two hours after infection, latency was established; we designated the corresponding infected cells K/iSLK-Vector, K/iSLK-RAB11FIP5, K/iSLK-WT and K/iSLK-RAB11FIP5^-/-^ cells. (B) K/iSLK-Vector and K/iSLK-RAB11FIP5 cells were induced with dox to stimulate lytic KSHV replication. Viral particles were labeled with the mouse anti-ORF65 antibody, while the trans-Golgi network was labeled with the rabbit anti-TGN46 antibody. FITC- and Cy3-conjugated secondary antibodies were used to visualize the labeled ORF65 and TGN46 proteins, respectively. (D) K/iSLK-WT and K/iSLK-RAB11FIP5^-/-^ cells were induced with dox to stimulate lytic KSHV replication. Viral particles were labeled with the mouse anti-ORF65 antibody, while the trans-Golgi network was labeled with the rabbit anti-TGN46 antibody. FITC- and Cy3-conjugated secondary antibodies were used to visualize the labeled ORF65 and TGN46 proteins, respectively.

ORF45, a tegument protein, associates with LRs and directs viral particles to the Golgi for release [29]. To evaluate the effect of RAB11FIP5 on the transport of viral particles to the trans-Golgi network, we isolated and expanded stable clones of iSLK-Vector, iSLK-RAB11FIP5, iSLK-WT and iSLK-RAB11FIP5^-/-^ cells from the monoclonal cell population by the limiting dilution method (Fig 8A and 8C). These cells were infected with stock KSHV obtained from BCBL1 cells (to generate K/iSLK-Vector, K/iSLK-RAB11FIP5, K/iSLK-WT and K/iSLK-RAB11FIP5^-/-^ cells) and were then reactivated by treatment with dox. Viral particles in cells were labeled with an antibody against the capsid protein ORF65 [29]. The result showed that RAB11FIP5 overexpression impaired the localization of the KSHV capsid to the trans-Golgi network (Fig 8B). However, knockout of RAB11FIP5 significantly promoted the localization of the KSHV capsid to the trans-Golgi network (Fig 8D). In contrast, overexpression of the RAB11FIP5 deletion mutant Δ16–127, which cannot bind to ORF45, had no significant effect on the localization of the KSHV capsid to the trans-Golgi network (S4 Fig). These data suggest that the RAB11FIP5 protein inhibits the transport of KSHV particles to the trans-Golgi network by interacting with the viral ORF45 protein.

## Discussion

KSHV release is crucial for its viral tumorigenicity and pathogenesis [13–16]. The commonly accepted concept of the process of herpesvirus release states that after capsids acquire the tegument, the tegument-capsid complex acquires its envelope by budding from Golgi network-derived membranes, and the mature virions are then secreted from the cells [57]. ORF45 is an outer tegument protein of KSHV that connects the capsid layer to the envelope layer and has been shown to participate in the regulation of viral particle release [4]. Previously, Zhu et al. constructed an ORF45-null recombinant virus and found that compared with wild-type virus, there was no differences in viral expression and lytic DNA replication, but the number of viral particles released was reduced by 10-fold [24]. ORF45 was reported to interact with the motor molecule kinesin-2 and promote the release of viral particles [28]. A recent study reported that ORF45 is associated with LRs in the Golgi apparatus and directs viral particles to the Golgi network for release [29]. Collectively, these findings indicate that ORF45 plays a vital role in events leading to the release of viral particles after viral reactivation, but the mechanisms regulating this process are incompletely understood. In the present study, the host RAB11FIP5 protein was found to interact with ORF45 and promote its lysosome-dependent degradation; thus, RAB11FIP5 inhibited ORF45-mediated release of KSHV particles. Our study reveals that a host protein inhibits virion production by antagonizing the function of the ORF45 protein, which further proves that ORF45 plays a key role in KSHV release and extends the understanding of host factors that mediate the KSHV release process.

Cells internalize extracellular signaling molecules through endocytosis, and endocytosed proteins are either recycled back to the plasma membrane or transported to lysosomes for degradation [30–32]. Endosomal transportation is largely regulated by the Ras-related in brain (RAB) family of small GTPases, which contains over 60 members with varying tissue distribution; individual RAB GTPases coordinate distinct steps in anterograde and retrograde protein trafficking [58,59]. RAB11 marks the major perinuclear endocytic recycling compartment that receives and transfers cargo back to the plasma membrane [34]. Jabe et al found that RAB11 preferentially targets Ca_v_1.2 for lysosomal degradation [37]. These findings suggest that RAB11 performs dual roles in endocytic recycling compartments. The RAB11FIP family comprises five proteins (RAB11FIP1-5) that are effectors of RAB11; these proteins interact with RAB11 and then regulate the RAB11-mediated endosomal recycling system [34]. A previous study confirmed that numerous viruses exploit the RAB11-mediated endosomal recycling system [43,44,47–50]. In this study, we elaborated on the detailed mechanism of RAB11FIP5 in regulating KSHV infection. We found that RAB11FIP5 interacted with the KSHV tegument protein ORF45, which plays a key role in regulating the release of KSHV particles. RAB11FIP5 overexpression in KSHV-infected cells significantly impaired virion release. In contrast, silencing endogenous RAB11FIP5 promoted virion release. Further study revealed that overexpression of RAB11FIP5 enhanced lysosomal translocation of ORF45 and increased its lysosome-dependent degradation. Moreover, we found that RAB11FIP5 was the only RAB11FIP member that interacted with KSHV ORF45 (S5 Fig). RAB11 may target ORF45 through RAB11FIP5 for lysosomal degradation under physiological conditions; alternatively, RAB11FIP5 may promote the translocation of ORF45 to lysosomes through other mechanisms requiring further exploration.

**Fig 9:**
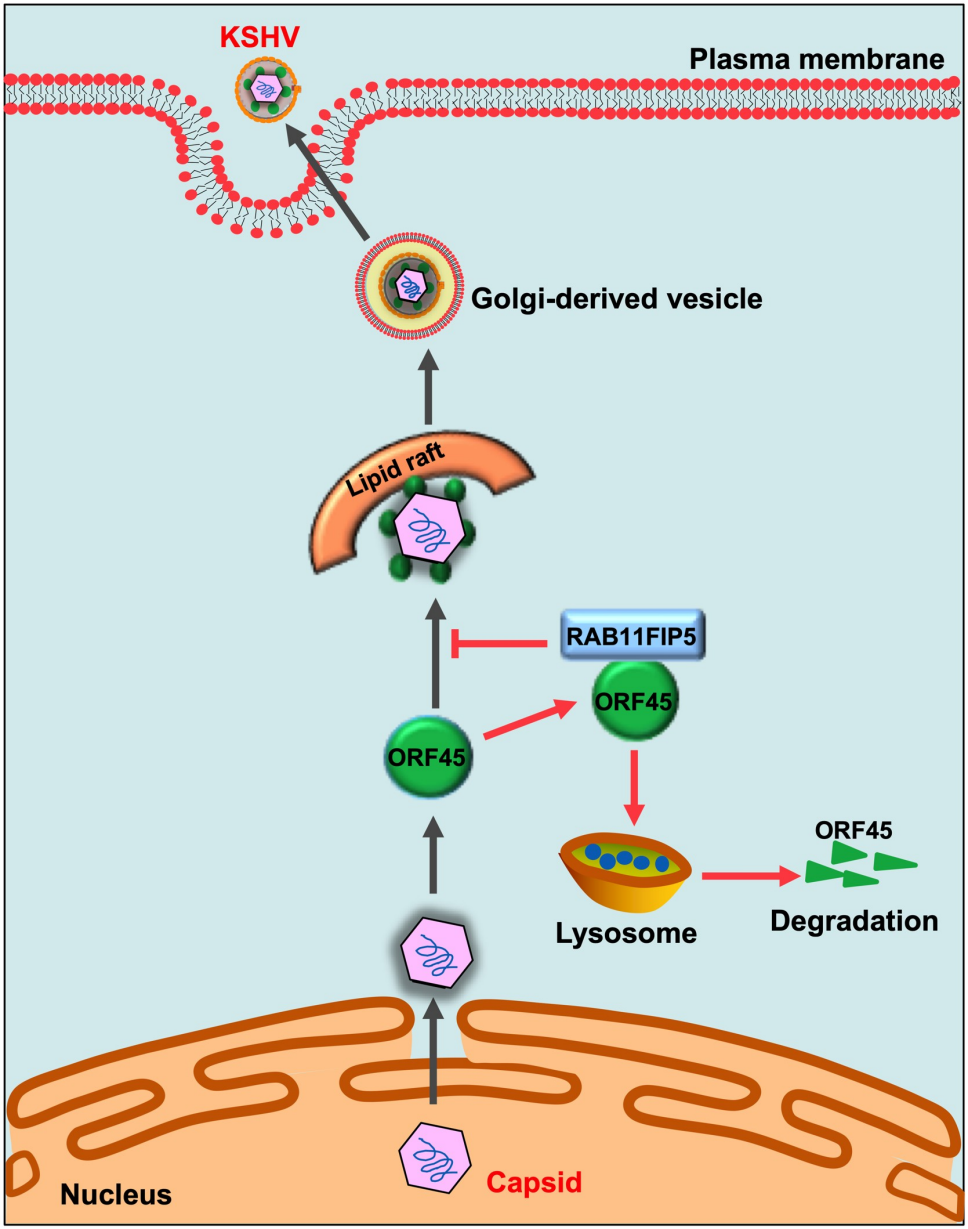
Working model indicating the role of RAB11FIP5 in ORF45-mediated release of KSHV particles. After budding into the cytoplasm, the tegument protein ORF45 directs tegumented capsids to LRs in the Golgi apparatus and promotes the release of KSHV particles. The host protein RAB11FIP5, a novel ORF45 binding protein, promotes the translocation of ORF45 to lysosomes and increases lysosome-dependent degradation of the ORF45 protein, which impairs the colocalization of ORF45 with LRs in the Golgi apparatus and then impairs the release of KSHV particles through Golgi transport vesicles.

In summary, our study reveals an important role of the host RAB11FIP5 protein in the process of ORF45-mediated release of KSHV particles (Fig 9). We found that RAB11FIP5 interacts with ORF45, which increases the translocation of ORF45 to lysosomes and promotes its degradation. In addition, RAB11FIP5 impairs the association of ORF45 with LRs in the Golgi apparatus and then reduces the transport of KSHV particles to the Golgi, which results in the inhibition of virion release. As RAB11FIP5 performs an important function in inhibiting ORF45-mediated virion release, promoting RAB11FIP5 expression in KSHV-targeted cells can be a potentially effective therapeutic approach for KSHV infection and related diseases.

## Materials and methods

### Cells and reagents

iSLK.RGB cells, which harbor wild-type BAC16.RGB were a kind gift from Jae Jung (University of Southern California, Los Angeles, USA) and were cultured in Dulbecco’s modified Eagle’s medium (DMEM, HyClone). The primary effusion lymphoma cell line BCBL1, which carries latently infected KSHV, was cultured in RPMI 1640 medium (HyClone). Human embryonic kidney 293T cells (HEK293T cells) and the Henrietta Lacks strain of cancer cells (HeLa cells) were purchased from the American Type Culture Collection (ATCC) and cultured in DMEM. The stable cell lines iSLK.RGB-Vector, iSLK.RGB-RAB11FIP5, HeLa-Vector and HeLa-RAB11FIP5 were established by infection with the indicated lentiviruses in accordance with the manufacturer’s instructions (System Bioscience, Palo Alto, USA). All cultures were supplemented with 10% FBS (Biological Industries) and 1% antibiotic solution (penicillin and streptomycin, Gibco) and grown at 37°C in a humidified environment supplemented with 5% CO_2_.

Dox, VPA and DNase I were purchased from Sigma-Aldrich (St. Louis, MO), as was an anti-Flag M2 affinity gel (Sigma-Aldrich, A2220). The proteasome inhibitor MG132 (474790) was purchased from Merck Millipore. The lysosomal inhibitor CHLO (C6628) was purchased from Sigma-Aldrich.

### Plasmids and antibodies

The RAB11FIP5 construct was amplified from an iSLK.RGB cell cDNA library, inserted into pCMV-HA at the EcoRI and KpnI sites, and subcloned into the pCDH-Flag (pCDH) vector at the XhoI and EcoRI sites and into the pGEX-4T vector at the EcoRI and XhoI sites. The ORF45 coding sequence was amplified from KSHV BAC16 genomic DNA and cloned into the pCDH vector at the XhoI and EcoRI sites. ORF45 was further subcloned into the expression vectors pCMV-HA and pET-30a. Flag-tagged truncation mutants of ORF45, including 115–407, 90–407, 77–407, 19–407, 1–332, 1–237, 1–114, Δ237–332 and Δ115–332 (see the schematics in Fig 3A and 3C), were amplified from the full-length template and cloned into the pCDH vector. pCDH expression plasmids containing the truncated RAB11FIP5 fragments, including 630–653, 1–490, 490–653, 16–127 and Δ16–127 (see the schematics in Fig 3E and 3G), were obtained by PCR amplification of pCDH-Flag-RAB11FIP5. All constructs were verified by DNA sequencing (Sangon Biotech Co., Ltd., Shanghai, China). A summary of the PCR primers used in this study is presented in Table 1.

**Table 1:** Primers used in this study.

Primer name	Purpose	Sequence of Oligonucleotide (5′-3′)
**pCMV-HA**		
RAB11FIP5	Full length	F CCGCTCGAGATGGCCCTGGTGCGGGG
	Cloning	R GGGGTACCCTATTTGGGGGGGCCCGGGG
ORF45	Full length	F CGGAATTCGGATGGCGATGTTTGTGAGGACC
	Cloning	R CCGCTCGAGGTCCAGCCACGGCCAGTTATAT
**pCDH-Flag**		
RAB11FIP5	Full length	F CCGCTCGAGATGGCCCTGGTGCGGGG
	Cloning	R CGGAATTCGATTTGGGGGGGCCCGGG
630–653	Truncated	F CCGCTCGAGATGATCGACCGGCTGCTGGT
	Cloning	R CGGAATTCGATTTGGGGGGGCCCGGG
1–490	Truncated	F CCGCTCGAGATGGCCCTGGTGCGGGG
	Cloning	R CGGAATTCGAGGGACCCCCCTTTTCCCCC
490–653	Truncated	F CCGCTCGAGATGATCCTGGGGGCCTCCCCACA
	Cloning	R CGGAATTCGATTTGGGGGGGCCCGGG
16–127	Truncated	F CCGCTCGAGATGTGGCTGCCCACGCAC
	Cloning	R CGGAATTCGAGGCCTGGCCCAGGAACTT
Δ16–127	Deletion	F ACGGTGGCGCTGGACGAGG
	Cloning	R GCGGGAAGGCCCCGCC
ORF45	Full length	F CCGCTCGAGATGGCGATGTTTGTGAGGACC
	Cloning	R CGGAATTCGAGTCCAGCCACGGCCAGTTATAT
115–407	Truncated	F CCGCTCGAGATGGAGGGGTACCCTGCAGACTT
	Cloning	R CGGAATTCGAGTCCAGCCACGGCCAGTTATATG
90–407	Truncated	F CCGCTCGAGATGGAATCTGAATATGACGAGGA
	Cloning	R CGGAATTCGAGTCCAGCCACGGCCAGTTATA
77–407	Truncated	F CCGCTCGAGATGCCTCTGGATCTACAGATATCCC
	Cloning	R CGGAATTCGAGTCCAGCCACGGCCAGTTATA
19–407	Truncated	F CCGCTCGAGATGCCAATTGAAGGAGCGC
	Cloning	R CGGAATTCGAGTCCAGCCACGGCCAGTTATA
1–332	Truncated	F CCGCTCGAGATGGCGATGTTTGTGAGGACC
	Cloning	R CGGAATTCGAGGATATAATTATCACGGACGCCAC
1–237	Truncated	F CCGCTCGAGATGGCGATGTTTGTGAGGACC
	Cloning	R CGGAATTCGAGATCGCGGTGGGTGCG
1–114	Truncated	F CCGCTCGAGATGGCGATGTTTGTGAGGACC
	Cloning	R CGGAATTCGATGGCTCGTCTTCCTCCTGA
Δ237–332	Deletion	F GATCGCGGTGGGTGCGGC
	Cloning	R TCGGGGAGTGACACAGACGAGGAG
Δ115–332	Deletion	F CTCTGGCTCGTCTTCCTCCTGAACATC
	Cloning	R TCGGGGAGTGACACAGACGAGGAG
GST-RAB11FIP5	PCR	F CGGAATTCATGGCCCTGGTGCGGGGCG
	Cloning	R CCGCTCGAGCTATTTGGGGGGGCCCGGGG
His-ORF45	PCR	F CGGAATTCATGGCGATGTTTGTGAGGACC
	Cloning	R CCGCTCGAGGTCCAGCCACGGCCAGTTATA
RAB11FIP5	qRT-PCR	F CAGTCTGAGCATAGCCCTGA
		R GTGGTAGTACTTGGCCGACT
ORF45	qRT-PCR	F CCTTTATCTCACTTGCGCCC
		R TCGTCGTCTGAAGGTGAGAG
RTA	qRT-PCR	F CGTGTAGAGATTCAACGGCG
		R AAGAGGTACCAGGTGTCGTG
PAN	qRT-PCR	F GCCGCTTCTGGTTTTCATTG
		R TTGCCAAAAGCGACGCA
ORF57	qRT-PCR	F GAGGTGTTTACGGACAGGGA
		R CCCACGTCATTTGTTCCTCC
ORF65	qRT-PCR	F ATGACTACGCTCACCATCCC
		R CGCCTTTGAATTCCACCCAT
LANA	qRT-PCR	F GCAGACTACACCTCCACACT
		R GTAGATCGGGGACTCTGTGG
K9	qRT-PCR	F GTCTCTGCGCCATTCAAAAC
		R CCGGACACGACAACTAAGAA
GAPDH	qRT-PCR	F AAATTGTCAGCAATGCCTCTTG
		R GGCATGGACAGTGGTCATAA

The following primary antibodies were used: anti-RAB11FIP5 rabbit monoclonal (ABclonal, A18142), anti-ORF45 mouse monoclonal (a kind gift from Yan Yuan, University of Pennsylvania, Philadelphia, Pennsylvania, USA), anti-RTA (prepared in our laboratory), anti-ORF64 (a kind gift from Yan Yuan, University of Pennsylvania, Philadelphia, Pennsylvania USA), anti-ORF65 (a kind gift from Shoujiang Gao, University of Pittsburgh, Pennsylvania, USA), anti-GST (ABclonal, AE001) anti-His (ABclonal, AE003), anti-Flag (Sigma-Aldrich, F1804), anti-HA (Sigma-Aldrich, H6908), anti-GAPDH (Sigma-Aldrich, G8795), anti-α-tubulin (Sigma-Aldrich, T6199), anti-LAMP1 (CST, #9091), anti-TGN46 (ProteinTech, 13573-1-AP), anti-caveolin-1 (ABclonal, A1555) and anti-TfR (ABclonal, A5865). The secondary antibodies used for western blotting were HRP-conjugated anti-mouse or anti-rabbit IgG (Jackson ImmunoResearch Laboratories). The secondary antibodies used for IFA were Alexa Fluor 488-conjugated goat anti-mouse/rabbit IgG and Alexa Fluor 555-conjugated goat anti-mouse/rabbit IgG, all of which were purchased from Thermo Fisher Scientific. Alexa Fluor 647-conjugated goat anti-rabbit IgG was purchased from Life Technologies.

### Immunoblot analysis and Co-IP

For immunoblot experiments, treated cells were lysed in lysis buffer (10 mM phosphate pH 7.4, 137 mM NaCl, 1% NP-40, 0.5% sodium deoxycholate, and 0.1% SDS) supplemented with 1 mM PMSF and protease inhibitor cocktail. Total or fractioned cellular extracts were mixed with 5× SDS gel loading buffer and resolved by SDS-PAGE. Cells were also treated with the lysosomal inhibitor CHLO at a final concentration of 50 μM or the proteasome inhibitor MG132 at a final concentration of 10 μM. For Co-IP, treated cells were lysed on ice in RIPA buffer for 30 min. A portion of the lysate was taken as the input, and the remaining lysate was used for immunoprecipitation with an anti-Flag antibody or an anti-ORF45 antibody. Membranes were blocked with 5% skim milk powder in TBST buffer for 1 h at room temperature and probed with the indicated primary antibodies overnight at 4°C. After hybridization with either goat anti-rabbit or goat anti-mouse secondary antibodies (diluted 1:5000) in TBST buffer, membranes were washed with TBST buffer four times (10 min each) before visualization with ECL reagents (GE).

### Immunofluorescence assay

HeLa cells were plated on coverslips in 24-well plates and transfected with the indicated plasmids. Twenty-four hours after transfection, cells were washed twice with PBS and fixed with 4% paraformaldehyde for 15 min. Cells were then permeabilized with 0.1% Triton X-100 for 15 min, blocked with 1% bovine serum albumin (BSA) in PBS for 30 min, and incubated with specific primary antibodies overnight at 4°C. After five washes with PBS containing 0.1% Tween 20, cells were incubated with FITC- or Cy3-conjugated secondary antibodies and 4′,6-diamidino-2-phenylindole (DAPI) for 1 h at room temperature. Finally, the coverslips were washed extensively and mounted on slides, which were visualized with a Leica TCS SF8 confocal microscope (Leica, Inc., Solms, Germany). Images were acquired with a digital camera and software (Leica, Inc.). To determine the localization of endogenous ORF45 and RAB11FIP5, we treated BCBL1 cells with VPA. Twelve hours after VPA treatment, cells were fixed and stained using antibodies specific for ORF45 and RAB11FIP5.

### GST pulldown assay

The GST-tagged RAB11FIP5 fusion protein and His-tagged ORF45 protein were expressed in *Escherichia coli* BL21(DE3) cells. Bacterial cells expressing the recombinant proteins were harvested and sonicated, and proteins were solubilized in PBS supplemented with PMSF and protease inhibitor cocktail. In the pulldown assay, bacterial lysates containing GST or GST-RAB11FIP5 were initially incubated with 30 μl of glutathione-Sepharose 4B for 4 h at 4°C with rotation. After the samples were washed, purified GST or GST-RAB11FIP5 protein was mixed with protein lysates containing His-ORF45 for another 6 h at 4°C and was then washed five times with RIPA buffer. Proteins pulled down by glutathione beads were extracted and analyzed by immunoblotting.

### RNA isolation and quantitative real-time PCR (RT-qPCR)

Total RNA was isolated from cells using TRIzol reagent (Invitrogen) following the manufacturer’s instructions. One microgram of RNA was used for reverse transcription with gDNA Eraser reverse transcription kits (Toyobo). cDNA was used for quantification of the indicated mRNA on a QuantStudio 6 Flex RT-PCR System (Applied Biosystems) with SYBR Green Real-Time PCR Master Mix kits (Toyobo) in accordance with the manufacturer’s instructions. Dissociation curve analysis of the products was conducted at the end of each PCR cycle to detect and validate the specific amplification of PCR products. The transcript level of each gene was normalized to that of GAPDH, and the 2^−ΔΔCT^ method was used to analyze gene expression in the samples. Data are presented as fold changes compared to the corresponding level in untreated control cells. The primer sequences used for RT-qPCR are listed in Table 1.

### Establishment and verification of stable cell lines

RAB11FIP5 overexpression lentiviruses were constructed based on the lentiviral vector pCDH-CMV-Flag-IRES-Blast. This RAB11FIP5 overexpression lentiviral vector and empty vectors were packaged in HEK293T cells by cotransfection with the Δ8.9 packaging plasmid and a plasmid expressing vesicular stomatitis virus G protein (pVSV-G) as described previously [60]. Two days later, the supernatant was collected and cleared by filtering through a 0.45 μm pore size filter. iSLK.RGB, HeLa and iSLK cell lines stably expressing RAB11FIP5 were generated by the addition of RAB11FIP5 stable expression and control lentiviral particles and centrifugation at 2500 rpm for 2 h. The medium was replaced with fresh DMEM. Forty-eight hours post infection (hpi), blasticidin (Sigma, 25 μg/ml) was added for selection of positive clones. Finally, the stable clones of HeLa-RAB11FIP5, HeLa-Vector, iSLK-RAB11FIP5 and iSLK-Vector cells were isolated and expanded from the monoclonal cell population by using the limiting dilution method. The expression of RAB11FIP5 was confirmed by western blotting.

### RNA interference

Two siRNA oligonucleotides targeting RAB11FIP5 and the corresponding negative control siRNA oligonucleotide were obtained from GenePharma. The sequences were as follows: siRAB11FIP5-#1, 5′-GGUACAAGCUGCACUCCAATT-3′; siRAB11FIP5-#2, 5′-GCAAGAUGGGCAAAGCCAATT-3′. The siRNA oligonucleotides were transfected into iSLK.RGB cells with Lipofectamine 2000 in accordance with the manufacturer’s instructions.

### Generation of RAB11FIP5-deficient cell lines

RAB11FIP5-deficient iSLK and HeLa cells were generated via the CRISPR/Cas9 system as described previously [61]. The single guide RNA (sgRNA) sequence targeting the human RAB11FIP5 gene (5’- CCTCGGTAGGTCTTGGACGG-3’) was cloned into the lentiCRISPRv2 vector and used to produce recombinant lentiviral vectors. iSLK cells and HeLa cells were transduced with sgRAB11FIP5 lentivirus or empty vector lentivirus (negative control). Forty-eight hours after transduction, blasticidin (25 μg/ml) was added for selection of positive clones. Finally, the stable clones acquired by using the limiting dilution method were expanded. Knockout of RAB11FIP5 was confirmed by western blotting.

### Quantification of viral genomic DNA

Intracellular viral genomic DNA and extracellular virion DNA were extracted from induced iSLK.RGB cells and cell culture supernatants, respectively. Supernatant (200 μl) from induced iSLK.RGB cells was collected and treated with DNase I (Sigma) for 1 h at 37°C. Intracellular and extracellular KSHV DNA were purified by a TIANamp Blood DNA Kit (Tiangen). The level of intracellular viral genomic DNA in each sample was normalized to that of GAPDH. The absolute copy number of the KSHV genome in the supernatant samples was determined by referencing a standard curve generated by qPCR on serial dilutions of a K9-encoding plasmid [62].

### Infection of HEK293T cells with progeny virus

Supernatant (500 μl) from each group was added to HEK293T cells in a 12-well plate and was then centrifuged at 2500 rpm for 2 h. The medium was replaced with fresh DMEM, and the cells were cultured for an additional 24 h. The infection rate of HEK293T cells was examined by fluorescence microscopy based on the RFP signal [60, 62].

### Lysosome isolation

HEK293T cells were transiently transfected with the ORF45 expression vector and RAB11FIP5 or empty vector. Cells were treated with CHLO for 6 h before lysosome isolation. Cell fractionation and lysosome isolation were performed with a lysosome isolation kit (BestBio, catalog # BB-31452, Shanghai, China) in accordance with the manufacturer’s instructions. Protein extracts were analyzed by western blotting with antibodies specific for the proteins of interest.

### Generation of stock KSHV

BCBL-1 cells (7×10^5^ cells/ml) latently infected with KSHV were induced with 1.5 mM VPA for 36 h [63,64]. The supernatant was collected, and debris was removed by centrifugation at 500 × g for 10 min and filtration through a 0.22 μm filter. Virions were concentrated by treatment with polyethylene glycol 6000 for 4 h [23]at 4°C, and each pellet obtained from 50 ml of supernatant was resuspended in 1 ml of serum-free RPMI 1640. This procedure yielded a 50-fold concentration of virions.

### Analysis of the effect of RAB11FIP5 on the localization of ORF45 and viral particles

To assess the effect of RAB11FIP5 on the lysosomal localization of ORF45, stable HeLa-Vector and HeLa-RAB11FIP5 cell lines were transfected with the ORF45 plasmid. Twenty-four hours after transfection, cells were treated with CHLO for 6 h and were then washed twice with PBS and fixed for 15 min in 4% paraformaldehyde. Cells were permeabilized with 0.1% Triton X-100 for 15 min, blocked with 1% BSA in PBS for 30 min, and incubated with mouse anti-ORF45 and rabbit anti-LAMP1 antibodies overnight at 4°C. After five washes with PBS containing 0.1% Tween 20, cells were incubated with FITC- or Cy3-conjugated secondary antibodies and DAPI for 1 h at room temperature.

To assess the effect of RAB11FIP5 on the localization of viral particles, stable clones of iSLK-Vector, iSLK-RAB11FIP5, iSLK-WT and iSLK-RAB11FIP5^-/-^ cells were infected with stock KSHV. Seventy-two hours post infection, the latency was established; we designated the corresponding infected cells K/iSLK-Vector, K/iSLK-RAB11FIP5, K/iSLK-WT and K/iSLK-RAB11FIP5^-/-^. These cells were treated with dox for 24 h and were then washed twice with PBS and fixed for 15 min with 4% paraformaldehyde. Cells were permeabilized with 0.1% Triton X-100 for 15 min, blocked with 1% BSA in PBS for 30 min, and incubated with mouse anti-ORF65 and rabbit anti-TGN46 antibodies overnight at 4°C. After five washes with PBS containing 0.1% Tween 20, cells were incubated with FITC- or Cy3-conjugated secondary antibodies and DAPI for 1 h at room temperature.

All slides containing cells were analyzed with a DM6000B fluorescence microscope (Leica, Inc., Solms, Germany).

### Membrane flotation assay

HEK293T cells were transiently transfected with the ORF45 expression plasmid and RAB11FIP5 or empty vector control. Forty-eight hours after transfection, cells were treated with CHLO for 6 h and were then harvested by scraping and pelleted by low-speed centrifugation in an Eppendorf centrifuge (4000 rpm for 3 min) at 4°C. Cells were then lysed on ice for 30 min in 2 ml of cold TNE buffer (50 mM Tris-HCl, 150 mM NaCl, and 5 mM EDTA) containing 1% Triton X-100. The cell lysates were centrifuged at 4000 rpm for 10 min at 4°C. Each clarified supernatant (2 ml) was mixed with 2 ml of 80% sucrose in TNE buffer containing 1% Triton X-100 to a final sucrose concentration of 40%. Subsequently, 3.66 ml of the mixture was placed at the bottom of the 12-ml ultracentrifuge tube and overlaid with 4.58 ml of 35% sucrose and 2.75 ml of 5% sucrose in TNE buffer containing 1% Triton X-100. Eleven (1 ml each) fractions were collected and subjected to trichloroacetic acid precipitation after centrifugation at 35,000 rpm for 16 h at 4°C in a P40ST rotor (Hitachi, Tokyo, Japan). The concentrated samples were mixed with SDS-PAGE loading buffer and were then boiled at 100°C for 10 min. The proteins in each layer were detected by western blotting with antibodies specific for the proteins of interest.

### Labeling and visualization of LRs with fluorescently labeled cholera toxin B subunit

Alexa Fluor 555 conjugated cholera toxin subunit B (CTB-555) (Life Technologies, 1:100 dilution) was used to label GM1-positive LRs [65] and to examine the colocalization of ORF45 with dynamic LRs [29] as previously described. Stable clones of HeLa-Vector and HeLa-RAB11FIP5 cells were transfected with the ORF45 plasmid. After 24 h of transfection, cells were treated with CHLO for 6 h and were then incubated with CTB-555 at 37°C for 30 min. Cells were fixed, permeabilized and labeled with mouse anti-ORF45 and rabbit anti-GM130 (ABclone) antibodies. Alexa Fluor 488-conjugated anti-mouse IgG and Alexa Fluor 647-conjugated anti-rabbit IgG (Life Technologies, 1:500 dilution) were used as the respective secondary antibodies.

### Statistical analysis

The results are expressed as the means ± SDs. Statistical analyses were performed on data from triplicate experiments by using Student’s t-test. A P value of < 0.05 was considered significant, and a P value of < 0.01 was considered highly significant (*P < 0.05; **P < 0.01; ***P < 0.001; ****P < 0.0001).

## Supporting information

S1 Fig Endogenous ORF45 and RAB11FIP5 are colocalized in iSLK.BAC16 cells. iSLK.BAC16 cells uninduced (Un) or induced with dox (In) were fixed and labeled with anti-RAB11FIP5 and anti-ORF45 antibodies and were then incubated with FITC- or Cy3-conjugated secondary antibodies. DAPI was used to label cell nuclei. Images of the colocalization sites were enlarged as shown.
(TIF)

S2 Fig Overexpression of the RAB11FIP5 mutant Δ16–127 has no effect on the release of progeny virus. (A) iSLK.RGB cells were stably transduced with lentiviruses containing a Flag-tagged RAB11FIP5 mutant Δ16–127 expression plasmid or an empty vector plasmid and named iSLK.RGB-Δ16–127 or iSLK.RGB-Vector cells, respectively. Overexpression of the RAB11FIP5 mutant Δ16–127 was detected by western blotting. (B) iSLK.RGB-Vector and iSLK.RGB-Δ16–127 cells were treated with dox for different time points as indicated. Extracellular virions were collected from the culture medium and treated with DNase I. Viral DNA was extracted, and KSHV genomic DNA copy numbers were estimated by qPCR by comparison with external standards containing known concentrations of the viral K9 plasmid.
(TIF)

S3 Fig RAB11FIP5 inhibits the release of KSHV progeny virions in BCBL1 cells. (A) BCBL1 cells were stably transduced with lentiviruses containing a Flag-tagged RAB11FIP5 expression plasmid or an empty vector plasmid and named BCBL1-RAB11FIP5 or BCBL1-Vector cells, respectively. The overexpression of RAB11FIP5 was detected by western blotting. (B) BCBL1-Vector and BCBL1-RAB11FIP5 cells were treated with VPA for different time points as indicated. Extracellular virions were collected from the culture medium and treated with DNase I. KSHV genomic DNA copy numbers were estimated as described above. (C) Lysates from VPA-treated BCBL1-Vector and BCBL1-RAB11FIP5 cells were analyzed by western blotting at the indicated time points. The expression levels of KSHV proteins, including ORF45 and RTA, were determined by immunoblotting with the indicated antibodies. (D) BCBL1 cells were transfected with control siRNA and siRAB11FIP5-#2. The knockdown efficiency was determined by western blotting. (E) BCBL1 cells were transfected with control siRNA and siRAB11FIP5-#2. Twenty-four hours after transfection, cells were induced with VPA for different time points as indicated. KSHV genomic DNA copy numbers were estimated as described above. (F) KSHV proteins, ORF45 and RTA, were determined by immunoblotting with the indicated antibodies.
(TIF)

S4 Fig RAB11FIP5 mutant Δ16–127 has no effect on the translocation of KSHV particles to the trans-Golgi network. (A) iSLK-BAC16 cells overexpressed RAB11FIP5 (iSLK-BAC16-RAB11FIP5) or empty vector (iSLK-BAC16-Vector). (B) iSLK-BAC16-Vector and iSLK-BAC16-RAB11FIP5 cells were induced with dox to stimulate lytic KSHV replication. Viral particles were labeled with the mouse anti-ORF65 antibody, while the trans-Golgi network was labeled with the rabbit anti-TGN46 antibody. FITC- and Cy3-conjugated secondary antibodies were used to visualize the labeled ORF65 and TGN46 proteins, respectively.
(TIF)

S5 Fig The interaction between ORF45 and five RAB11FIP family members. HEK293T cells were cotransfected with Flag-ORF45 and HA-RAB11FIP1, HA-RAB11FIP2, HA-RAB11FIP3, HA-RAB11FIP4 or HA-RAB11FIP5. Cell lysates were immunoprecipitated with an anti-Flag antibody and were then analyzed by western blotting with the indicated antibodies.
(TIF)
