# Supplementary figures and images for: Host RAB11FIP5 protein inhibits the release of Kaposi’s sarcoma-associated herpesvirus particles by promoting lysosomal degradation of ORF45
Source: PLoS Pathog. 2020 Dec 14;16(12):e1009099. doi: 10.1371/journal.ppat.1009099 (PMC7735600; doi:10.1371/journal.ppat.1009099)

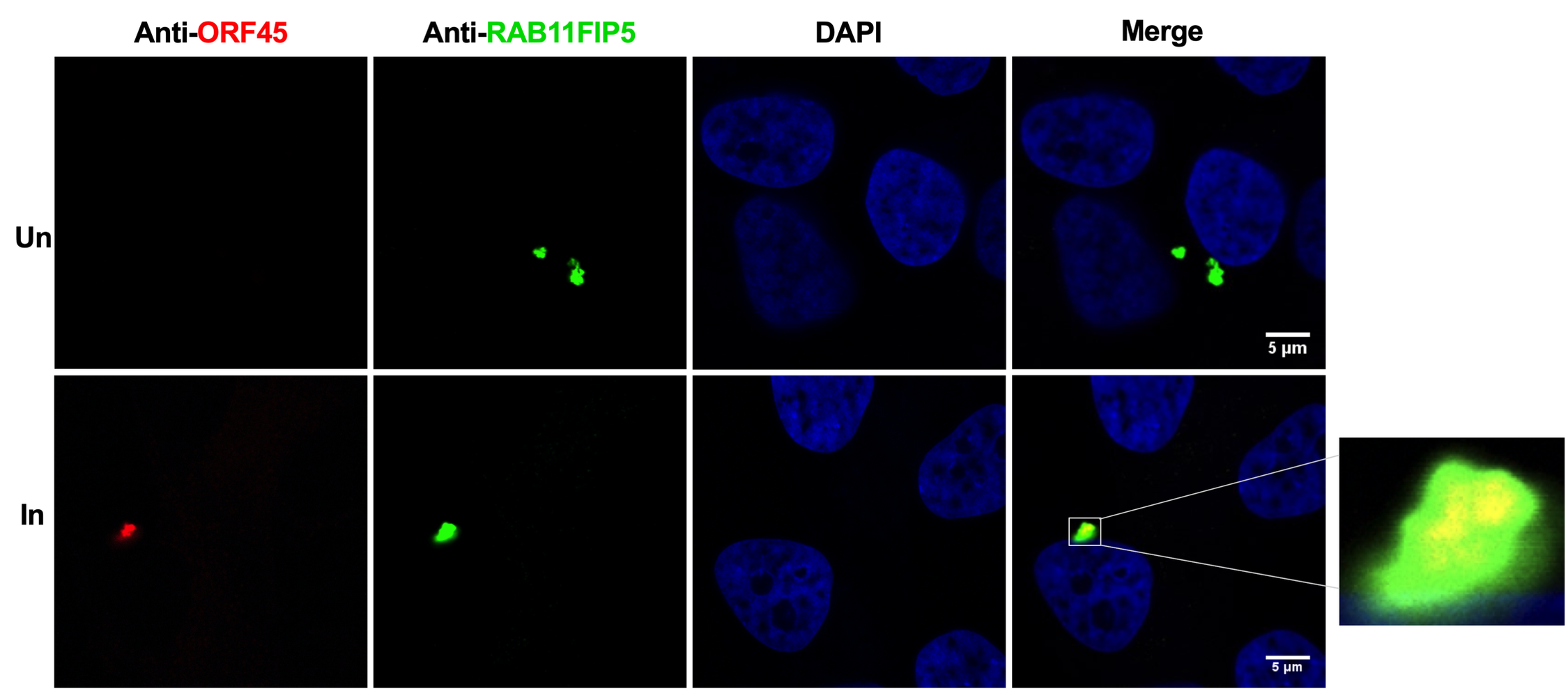

Supplement: S1 Fig — iSLK.BAC16 cells uninduced (Un) or induced with dox (In) were fixed and labeled with anti-RAB11FIP5 and anti-ORF45 antibodies and were then incubated with FITC- or Cy3-conjugated secondary antibodies. DAPI was used to label cell nuclei. Images of the colocalization sites were enlarged as shown. (TIF) [file ppat.1009099.s001.tif]

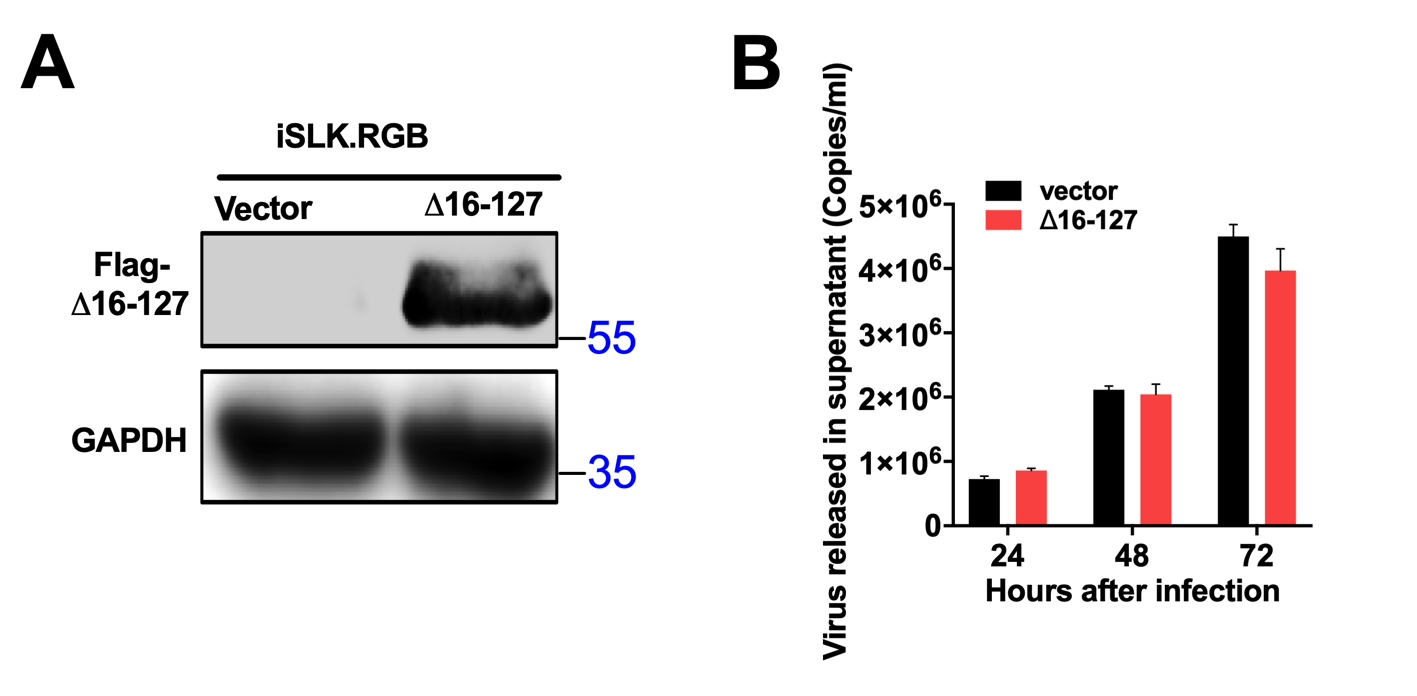

Supplement: S2 Fig — (A) iSLK.RGB cells were stably transduced with lentiviruses containing a Flag-tagged RAB11FIP5 mutant Δ16–127 expression plasmid or an empty vector plasmid and named iSLK.RGB-Δ16–127 or iSLK.RGB-Vector cells, respectively. Overexpression of the RAB11FIP5 mutant Δ16–127 was detected by western blotting. (B) iSLK.RGB-Vector and iSLK.RGB-Δ16–127 cells were treated with dox for different time points as indicated. Extracellular virions were collected from the culture medium and treated with DNase I. Viral DNA was extracted, and KSHV genomic DNA copy numbers were estimated by qPCR by comparison with external standards containing known concentrations of the viral K9 plasmid. (TIF) [file ppat.1009099.s002.tif]

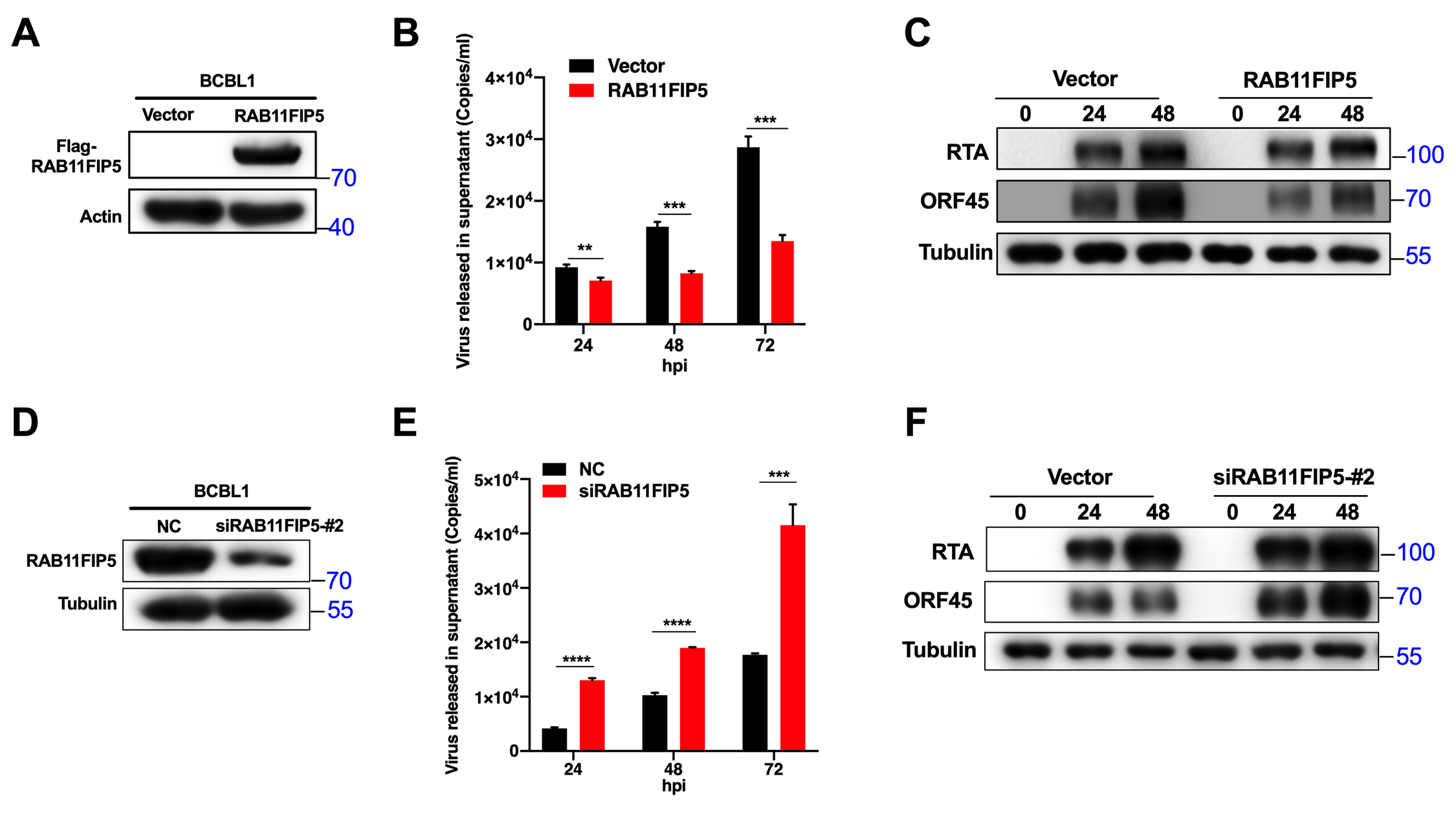

Supplement: S3 Fig — (A) BCBL1 cells were stably transduced with lentiviruses containing a Flag-tagged RAB11FIP5 expression plasmid or an empty vector plasmid and named BCBL1-RAB11FIP5 or BCBL1-Vector cells, respectively. The overexpression of RAB11FIP5 was detected by western blotting. (B) BCBL1-Vector and BCBL1-RAB11FIP5 cells were treated with VPA for different time points as indicated. Extracellular virions were collected from the culture medium and treated with DNase I. KSHV genomic DNA copy numbers were estimated as described above. (C) Lysates from VPA-treated BCBL1-Vector and BCBL1-RAB11FIP5 cells were analyzed by western blotting at the indicated time points. The expression levels of KSHV proteins, including ORF45 and RTA, were determined by immunoblotting with the indicated antibodies. (D) BCBL1 cells were transfected with control siRNA and siRAB11FIP5-#2. The knockdown efficiency was determined by western blotting. (E) BCBL1 cells were transfected with control siRNA and siRAB11FIP5-#2. Twenty-four hours after transfection, cells were induced with VPA for different time points as indicated. KSHV genomic DNA copy numbers were estimated as described above. (F) KSHV proteins, ORF45 and RTA, were determined by immunoblotting with the indicated antibodies. (TIF) [file ppat.1009099.s003.tif]

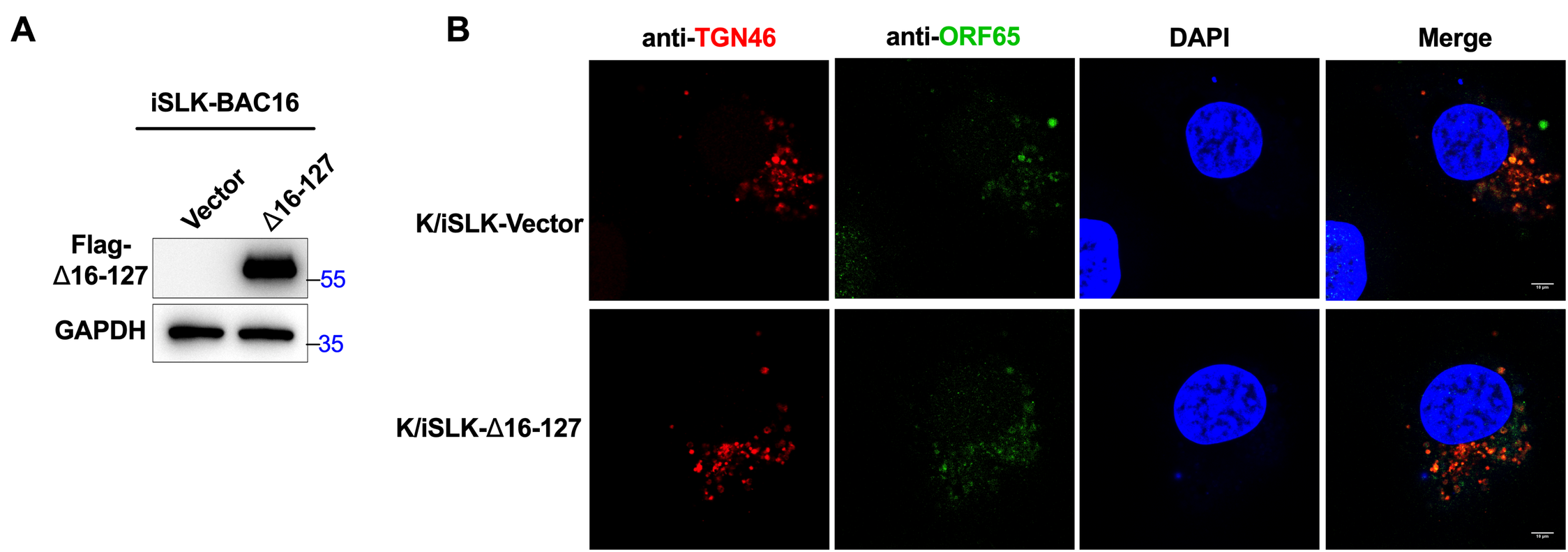

Supplement: S4 Fig — (A) iSLK-BAC16 cells overexpressed RAB11FIP5 (iSLK-BAC16-RAB11FIP5) or empty vector (iSLK-BAC16-Vector). (B) iSLK-BAC16-Vector and iSLK-BAC16-RAB11FIP5 cells were induced with dox to stimulate lytic KSHV replication. Viral particles were labeled with the mouse anti-ORF65 antibody, while the trans-Golgi network was labeled with the rabbit anti-TGN46 antibody. FITC- and Cy3-conjugated secondary antibodies were used to visualize the labeled ORF65 and TGN46 proteins, respectively. (TIF) [file ppat.1009099.s004.tif]

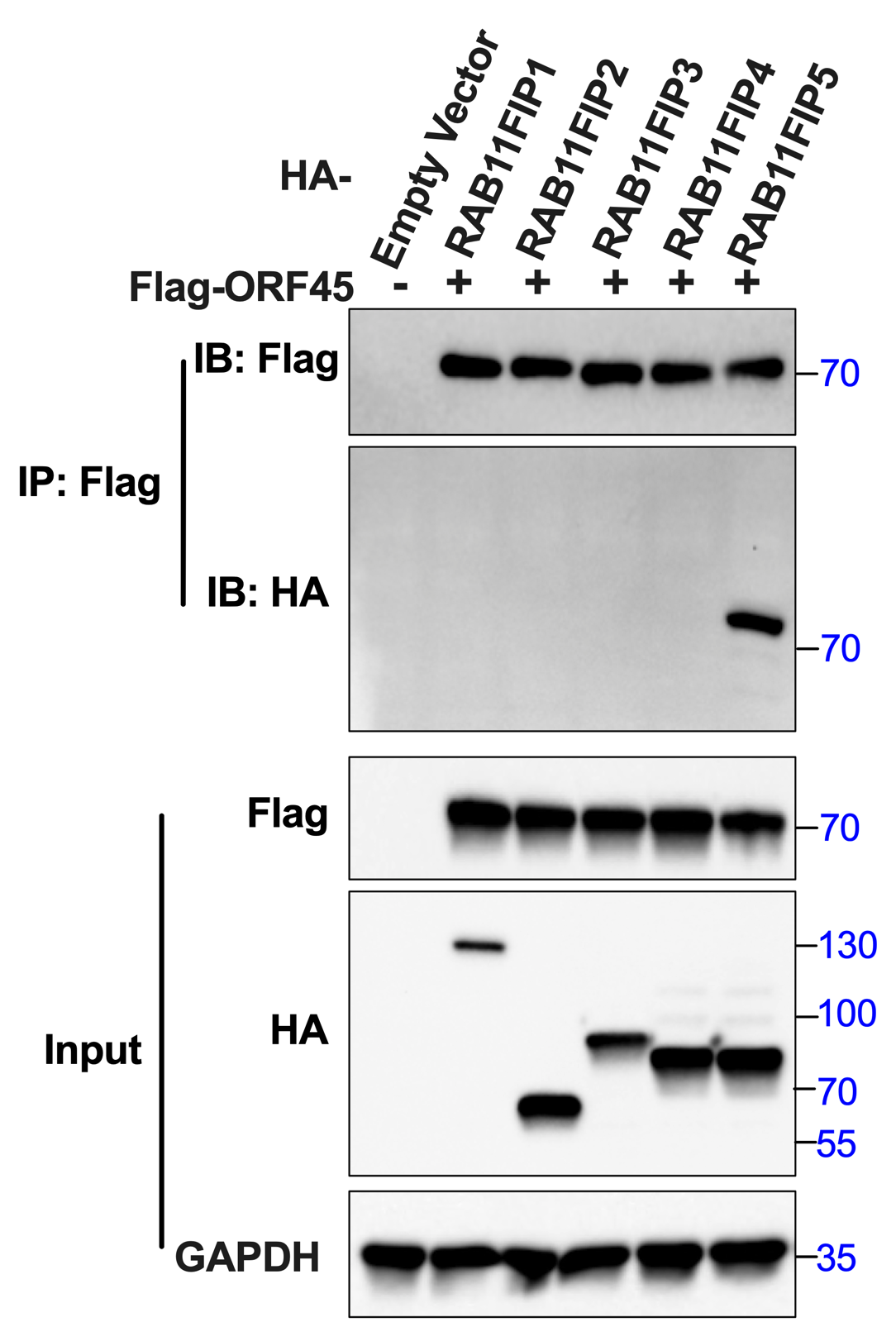

Supplement: S5 Fig — HEK293T cells were cotransfected with Flag-ORF45 and HA-RAB11FIP1, HA-RAB11FIP2, HA-RAB11FIP3, HA-RAB11FIP4 or HA-RAB11FIP5. Cell lysates were immunoprecipitated with an anti-Flag antibody and were then analyzed by western blotting with the indicated antibodies. (TIF) [file ppat.1009099.s005.tif]
